# Supplementary material for: Unveiling molecular interactions that stabilize bacterial adhesion pili
Source: Biophys J. 2022 Apr 30;121(11):2096–106. doi: 10.1016/j.bpj.2022.04.036 (PMC9247471; doi:10.1016/j.bpj.2022.04.036)
Supplement: Document S2. Article plus supporting material [file mmc6.pdf]

Biophysical Journal
Article

Biophysical Society

Unveiling molecular interactions that stabilize bacterial adhesion pili

Tobias Dahlberg,[1] Joseph L. Baker,[2] Esther Bullitt,[3,*] and Magnus Andersson[1,4,*]
[1]Department of Physics, Umeå University, Umeå, Sweden; [2]Department of Chemistry, The College of New Jersey, Ewing, New Jersey; [3]Department of Physiology & Biophysics, Boston University School of Medicine, Boston, Massachusetts; and [4]Umeå Centre for Microbial Research (UCMR), Umeå, Sweden

ABSTRACT Adhesion pili assembled by the chaperone-usher pathway are superelastic helical filaments on the surface of bacteria, optimized for attachment to target cells. Here, we investigate the biophysical function and structural interactions that stabilize P pili from uropathogenic bacteria. Using optical tweezers, we measure P pilus subunit-subunit interaction dynamics and show that pilus compliance is contour-length dependent. Atomic details of subunit-subunit interactions of pili under tension are shown using steered molecular dynamics (sMD) simulations. sMD results also indicate that the N-terminal "staple" region of P pili, which provides interactions with pilins that are four and five subunits away, significantly stabilizes the helical filament structure. These data are consistent with previous structural data, and suggest that more layer-to-layer interactions could compensate for the lack of a staple in type 1 pili. This study informs our understanding of essential structural and dynamic features of adhesion pili, supporting the hypothesis that the function of pili is critically dependent on their structure and biophysical properties.

SIGNIFICANCE Bacteria express micrometer-long adhesion pili optimized for attachment in different environmental niches. A specific class of adhesion pili is assembled from subunits into helix-like structures that exhibit superelastic behavior important for initial and sustained attachment when exposed to fluid flow. In this work, we investigate the network of subunit interactions leading to superelasticity using force-measuring optical tweezers and molecular dynamics simulations. We reveal subunit dynamics and the interactions most critical for pilus stability. This study illuminates essential features found in a class of adhesion pili important in many diseases and adds momentum to the observation that bacterial adhesion is supported by specialized biophysical properties of pili.

INTRODUCTION

Many bacteria express micrometer-long surface fibers called adhesion pili (or fimbriae), which are key virulence factors that mediate host-pathogen interactions. For Gram-negative bacteria, adhesive pili class are most commonly assembled via the classical chaperone-usher (CU) pathway (1). P pili are an archetypal CU pilus encoded by the *pap* gene, which is significantly prevalent among strains of uropathogenic *Escherichia coli* (UPEC) that cause pyelonephritis (kidney inflammation) (2,3). P pili are assembled from approximately 1000 identical protein subunits (PapA) into an 8-nm thick helically wound rod with a short tip fibrillum composed of minor pilins PapF, PapE, and PapK. The fibrillum is located at the pilus distal end (4), with the adhesin protein PapG located at the very tip. This adhesin is a lectin that binds to galabiose-containing glycosphingolipids (5).

To assemble a P pilus, all subunits (pilins) are transported from the inner membrane through the periplasm via the general secretory pathway (1). During their transport to the outer membrane by the PapC usher (6), the pilins are folded and stabilized by the periplasmic chaperone PapD via donor-strand complementation (DSC) (7). At the outer membrane, each pilin subunit is transferred from the chaperone to the usher, where it binds to the linear polymer of previously assembled subunits via donor-strand exchange (DSE). As the polymer is assembled, subunits are translocated to the cell surface through the usher's central pore (1). After exiting the pore, the PapA polymer forms a quaternary helical surface filament of 3.28 subunits per turn with a pitch of 25.2 Å and a diameter of 81 Å (8,9). Each subunit n in the helical filament is bound through

Submitted January 20, 2022, and accepted for publication April 27, 2022.

*Correspondence: bullitt@bu.edu or magnus.andersson@umu.se

Tobias Dahlberg and Joseph L. Baker contributed equally to this work.

Editor: David Sept.

https://doi.org/10.1016/j.bpj.2022.04.036

© 2022 Biophysical Society.

This is an open access article under the CC BY license (http://creativecommons.org/licenses/by/4.0/).

hydrophobic and weak hydrophilic interactions with 10 other subunits, that is, with five preceding (−5 to −1) and five succeeding (+1 to +5) subunits, forming a large network of interactions (Fig. 1). The extension of this network to such distant subunits in P pili (n to $n-4$ and $n-5$) is primarily due to the staple that is composed of the first seven residues of the PapA N terminus. It is therefore hypothesized that the staple has an extensive stabilizing role despite the fact that the staple region might not be essential in rod formation (9). Interestingly, so far, the staple region has only been found on P pili and is missing in closely related UPEC pili such as type 1. Also, this staple region is missing in the archetypal CFA/I pili expressed by enterotoxigenic *Escherichia coli* (ETEC) (10).

Despite the large interaction network of subunits, the bulk of the surface contact area is between the nth subunit and −3 and +3 (Fig. 1) (9). Not surprisingly, the magnitude of the surface contact area is closely related to the force needed to uncoil the quaternary structure of helical pili. For example, from cryoelectron microscopy (cryo-EM) structures and force-extension experiments, it has been shown that PapA subunits have a buried surface area between n and $n+3$ of 1453 $Å^2$ and P pili require 28 pN of force to uncoil, whereas FimA in type 1 pili and CfaB in CFA/I pili have 1615 $Å^2$ and 1087 $Å^2$ and require 30 pN and 7 pN of force to uncoil, respectively (9–12). The experimentally measured forces needed to uncoil pili are of the same magnitude as the fluid flow forces expected to be present in the in vivo environmental niches of UPEC bacterial cells (13). For example, in the proximal renal tubule, urine flow exposes cells to 0.017 pN/ μm^2 shear stress and, in the urethra, shear stress can reach 0.3–0.5 pN/ μm^2 (14). We note that this low shear stress is due to the proximity of bacteria to the epithelial cell wall, where the fluid flow velocity is much lower than the flow velocity at the tube center. Uncoiling of pili at these levels of force is further supported by in vitro fluid flow experiments, which showed that type 1 pili expressed on *E. coli* cells uncoil when exposed to 0.72 pN/ μm^2 shear stress (15). The ability of pili to uncoil by breaking sequential layer-to-layer interactions reduces the shear forces on the pili, and is thereby expected to aid sustained bacteria adhesion under fluid flow (16,17). This hypothesis is supported by in vivo data in which bacteria expressing mutant type 1 pili, with reduced surface contact area between layer-to-layer subunits, had a significantly reduced ability to cause intestinal colonization and bladder infection in mice (18).

Thus, the specific biophysical properties of pili are crucial for bacterial adhesion. Over the years, numerous helical filament pilus types have been characterized. These measurements revealed that pili are elastic and superelastic filaments and that they can be completely uncoiled into a linear fiber under force. When the force is removed, the structure can regain its helical quaternary shape (12,19–27). Although several studies have been performed and models developed to explain the superelasticity behavior, there are still several open research questions that need attention. For example, does the staple found in

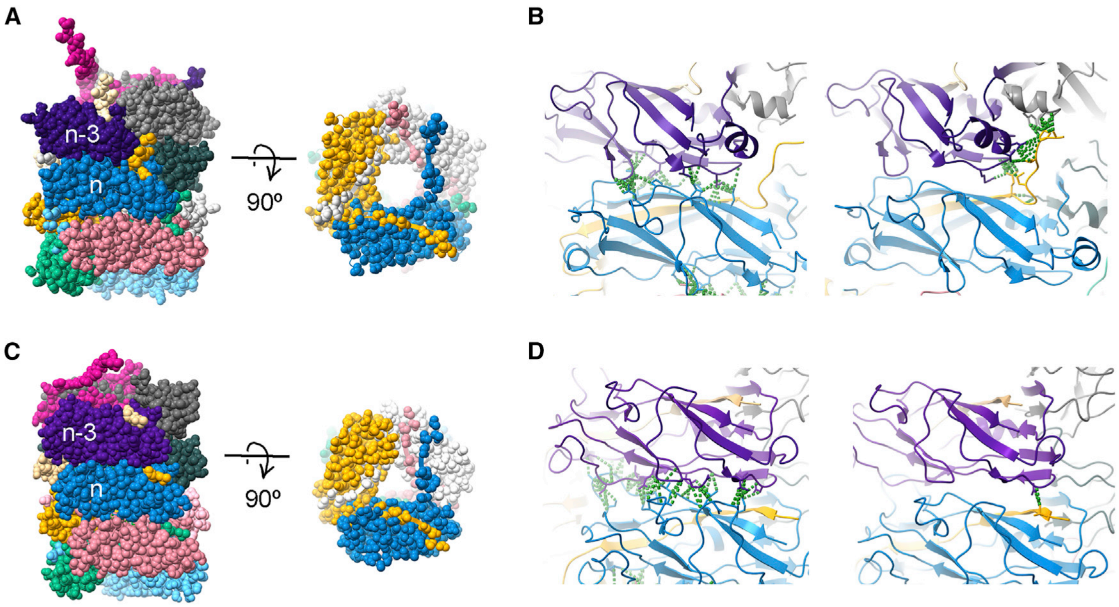

FIGURE 1 The staple region of P pili provides specialized subunit-subunit interactions. (*A*) A surface view of P pili with subunits individually colored; the N-terminal extension of the $n+1$ yellow subunit is inserted into a groove in the nth subunit and has a staple that forms contacts with subunits $n-3$ and $n-4$. (*B*) Contacts between P pili subunits are shown in green between subunits n and $n-3$ (*left*, layer-to-layer interactions) and between subunit $n+1$ to $n-3$ and $n-4$ (*right*, staple interactions). (*C*) Type 1 pilus structure, for comparison with (*A*). (*D*) Type 1 pili have increased n to $n-3$ interactions (*left*) and only one contact of $n+1$ to $n-3$, as there is no staple region; cf (*B*). To see this figure in color, go online.

P pili subunits provide a stabilizing role when tensile stress is applied, and are there other possible functions? How do subunits re-orientate when unbound? How much longer does a pilus get upon extension? This number varies in the literature. While the extension response of a pilus is well described by biophysical models, these models treat subunits as individual blocks connected by springs and do not consider individual molecular interactions. Therefore, questions about the contact network between pilins are still unanswered. For example, what contacts are most important for pilus stability? How fast are the transitions between the subunits' bound and unbound states? Are there intermediate or alternative states that the subunits can visit during binding and unbinding? To answer these research questions, we combined optical tweezers (OT) force experiments and molecular dynamics (MD) simulations to unveil the molecular interactions that stabilize CU pili and their role for pilus mechanical and kinetic properties.

MATERIALS AND METHODS

Bacterial strains and growth conditions

We used the *E. coli* strain HB101 as host strain for the plasmid pHMG93 to express UPEC-related P pili (28–30). We cultured the bacteria on trypticase soy agar at 37°C for 24 h.

OT force measurements

To apply strain and thereby extend a pilus and track its length fluctuations, we used an in-house-built OT setup. The OT setup uses an inverted microscope (Olympus IX71, Olympus, Japan) as a base. To image the sample and form the trap, we used a water immersion objective (model: UPlanSApo60XWIR 60X N.A. = 1.2; Olympus, Japan) and a 1920 × 1440 pixel CMOS camera (model: C11440-10C, Hamamatsu) (31). We minimized the amount of noise in the OT setup by using Allan variance analysis (32), and we used an active power spectrum method to calibrate the trap (33). During calibration, we oscillated the bead at 32 Hz with an amplitude of 100 nm. We sampled the microbead position at 131,072 Hz and averaged 32 consecutive datasets acquired for 0.25 s each. In general, the trap stiffness was 370 pN/μm. An example of a power spectrum with a corresponding fit from our instrument is shown in Fig. S1.

To extend a pilus, we first attached a bacterium to a poly-L-lysine-coated immobilized microsphere, and then trapped a bead with our optical trap. The trapped bead was moved in proximity to the bacterium to attach a pilus. Thereafter, we moved the piezo stage at a speed of 50 nm/s, below steady state, to apply tensile fore. To measure the kinetics of subunit opening we uncoiled 150 nm of the rod and then kept the piezo stage and trap stationary. The measurement procedure is shown in Fig. 2 *A*. We sampled the force and position of the bead at 100 kHz with an anti-aliasing filter set to 50 kHz. We recorded five data series of 30 s each for a total of 2.5 min of data per pili. We show a schematic with details of the setup and more information on the measurement procedure in section S0 and Fig. S2 in supporting material.

Force data analysis

We averaged the time-series data to an effective sampling rate of 500 Hz to remove thermal noise and increase spatial resolution. As we measured using a constant trap position, instead of constant force, the measured displacements of the bead did not reflect the true length changes of the pili. To correct for this effect, we did compliance corrections for each dataset using the stiffness of the pilus. For details on how we retrieved the stiffness from variance of the bead fluctuations and did the compliance correction, see section S1 in supporting material. As the bead is connected to the trap and pilus in parallel, their stiffness is additive, so that difference of the stiffness when attached to the pilus and that for the bead in the trap alone gives an estimate of the stiffness of the pilus. With this estimated stiffness, we generally determined a correction factor of approximately 1.5, indicating a stiffness of approximately 700 pN/um. We then analyzed the corrected data by taking a sliding window histogram with a window length of approximately 1 s of the data to retrieve the distance between states.

MD simulations

The initial structure of the *E. coli* P pilus was obtained from PDB entry 5FLU (9). Simulations were carried out for a 7mer segment of the filament following a protocol very similar to (11) but with some changes made related to the larger system size simulated here. The program Amber20 was used to perform all simulations for this work (34). Steered MD (sMD) simulations were carried out at constant velocities of 1 Å/ns and 5 Å/ns with the staple region of the PapA N-terminal extension present, and also with amino acids in the staple region removed. In each case (each pulling speed and with/without the staple region) five simulations were performed. Filament extension in the sMD simulations was directed along the z axis, which is aligned with the filament axis. For additional details, please see the description in the supporting material.

RESULTS AND DISCUSSION

Force-extension experiments unveil the kinetics of subunit opening and a contour-length-dependent compliance

To study the layer-to-layer interactions that stabilize the P pilus rod and the consequences of bond breakage for pili mechanics, we used OT. OT measurements, and to some extent atomic force measurements, have shown that helix-like pili often exhibit two or three different modes of elongation, which show up as distinct regions in their force-extension responses (21,35). These regions are well described by elastic and entropic elasticity (region I), superelasticity (region II), and a combination of elastic and entropic elasticity with a phase transition (III) (Fig. 2 *B*) (11,36). In Fig. 2 *B*, we can see that the initial force response (region I) of a P pilus continues linearly until it reaches a threshold force of 28 pN. At 28 pN, we enter region II, where layer-to-layer interactions break, resulting in a sequential uncoiling of the P pilus rod. Due to the tight native packing of the pili, 3.28 subunits per turn (8), the sequential uncoiling causes the pilus to elongate (21), as illustrated in Fig. 2 *C* (panel I), keeping the force experienced by the pilus close to constant.

However, although the force in region II appears constant, it is rapidly fluctuating due to thermal energy that is breaking and reforming the layer-to-layer interactions that bind the subunits in a coiled formation. That is, subunits randomly shift between their bound and unbound state. As such, we would expect a pilus held at a constant force in region II to undergo random length changes over time. To

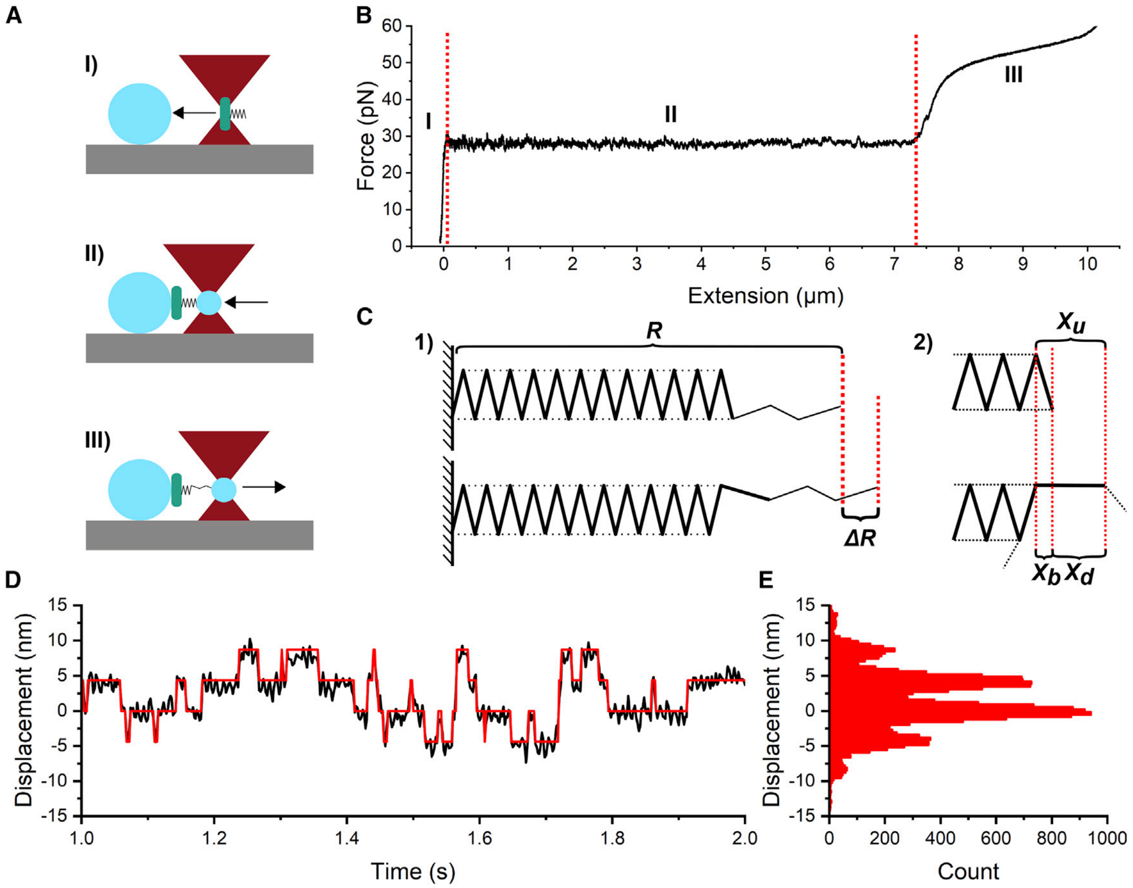

FIGURE 2 Force-extension experiments show that P pili uncoil in a step-wise manner. (*A*) An illustration of a force-extension measurement. I) We trap and mount a single bacterium (*teal*) to a large poly-L-lysine-coated microsphere (*sky blue*) fixed to the sample chamber. II) We trap and attach a microsphere to a pilus (*black*) on the bacterium. III) We separate the bacterium and trapped microsphere, thus applying tensile force to the pilus and forcing it to extend. By stopping at a given position we can also monitor how layer-to-layer interactions break and re-form under steady-state conditions. (*B*) An example of a force-extension curve of a P pilus. The different regions are marked with I, II, and III. Boundaries between the regions are demarcated by dashed red lines. (*C*) 1) Illustration showing the helical structure of a P pilus with a total end-to-end distance R made out of many bound subunits (*solid lines*) in which layer-to-layer interactions (*dashed lines*) can break and reform, causing an end-to-end distance change ΔR. 2) Zoomed-in view of the layer-layer-interaction breaking showing the contour-length contribution of a bound (folded) subunit X_b, the contribution of an unbound subunit X_u, where X_d is the difference in pilus contour length after a subunit uncoils. (*D*) An excerpt from a time series showing the displacement of the trapped microbead. The displacement states are shown in red. (*E*) Displacement histogram of the time-series data showing discrete peaks due to the changing number of bound and unbound subunits. To see this figure in color, go online.

study these length changes, we extended the pilus and uncoiled it roughly 150 nm into region II, and then held the bead stationary. We stopped at 150 nm of extension since pili get softer as they uncoil, reducing temporal and spatial resolution in our measurements; a dataset showing this softening and the change in the distribution of fluctuations of a pilus measured at two positions along region II is shown in Fig. S3. Therefore, we measured the random length changes of pili that were mostly in the helical, coiled arrangement with only a small region of the pilus uncoiled. Using this procedure, we obtained a time series of small length changes in the trapped pilus. For these experimentally measured displacements to reflect the pilus's end-to-end length changes (R in Fig. 2 *C*), we included a compliance correction. This compliance correction accounts for the effects of the finite trap stiffness; see section S1 in supporting material for details on the compliance correction. We show an example of a 2-s section from a time series in Fig. 2 *D*. These data show clear random transitions between discrete displacement states corresponding to the varying number of subunits in the bound or unbound state.

To analyze these state transitions in detail, we plotted a histogram of the measured length fluctuations, as shown in Fig. 2 *E*. This histogram shows that the pilus mostly visits six discrete states, corresponding to six subunits randomly being in the bound or unbound state. Further, we find that the spacing between these states is 4.2 ± 0.2 nm (n = 10,000 transitions, 10 bacteria, two biological replicates). This value corresponds to the change in end-to-end length of the pilus when a single subunit switches from the bound state to the unbound state, shown as ΔR in Fig. 2 *C* (panel I). However, as the uncoiled region of the

pilus is highly flexible (2 nm thick), thermal fluctuations will contract it due to conformational entropy. Thus, the mean contribution of a subunit to the total end-to-end distance will be less than its contribution to the length of the pilus backbone, the contour length. For these distance definitions, see Fig. S4. To relate the change in contour length to the measured end-to-end distance change, we created an entropic elasticity model for a partially uncoiled pilus, as described in section S1 of supporting material. Our entropic elasticity model combines a worm-like chain model and a free-jointed-chain model that describes the coiled and uncoiled part, respectively. Comparing this model with a previous model (36) shows a significantly better agreement with experimentally measured pilus stiffness (Fig. S5). Using this new stiffness model, we find that the increased contour length contributed by a single subunit going from the bound to the unbound state is 4.35 $\pm$ 0.2 nm, corresponding to the contour length increase of the pilus after a subunit unbinds, X_d in Fig. 2 *C* (panel II).

We can relate this value, X_d, to the length contributed by a single subunit bound within the coiled region, X_u, through the relationship $X_u = X_d + X_b$. The contour-length contribution X_b of a bound subunit is known from the molecular structure and is 0.754 nm (37). Thus, we can estimate X_u to be $\sim$ 5.1 nm (4.35 + 0.75 nm). This value is close to the $\sim$ 5-nm length of a PapA subunit (38). Thus, we can infer that the donor strand, which acts as a hinge between the subunits, does not restrict subunit movement during uncoiling, allowing the pilus to become fully linearized.

Knowing this, we can also estimate the pilus elongation ratio, the ratio between its coiled and uncoiled length. We determine this by taking the ratio of a subunit's bound and unbound length. Thus, the elongation ratio is given by (5.1 $-$ 0.75)/0.75 = 5.8, which means that a P pilus gets roughly six times longer after uncoiling. This elongation ratio lands in the middle of the previously reported values for P pili, which reported four to seven times elongation (8,19,21). Further, it is worth noting that our value of X_d is larger than previously reported for P pili, where a change in end-to-end distance was assessed to 3.5 nm (21,39). Most likely, this difference originates from the simple geometrical and kinetic models used in that work to describe the uncoiling of pili.

In addition, we see that the histogram in Fig. 2 *E* indicates no significant intermediate states between the bound and unbound states in our force experiments. To ensure that we did not miss any fast transitions due to a low sampling rate, we increased our sampling rate from 500 Hz to 100 kHz, the limit of our system. However, even sampling at this speed did not indicate additional intermediate steps. Therefore, if there are intermediate states between the bound and unbound states of a subunit, they must exist on a shorter time scale than the millisecond response time of our setup. Further, it is worth noting that we do not see any indication of P pili recoiling at two force levels, as is seen in some force curves of type 1 and SII pili (22,40,41). In particular, for type 1 pili, this observation might reflect that the rod can adopt two different folding configurations of the quaternary structures, as observed when comparing type 1 pili assembled either in vitro or in vivo (42,43).

It is also worth noting that, in relation to type 1 pili, we see that the length contributed by a single P subunit, $\sim$ 4.3 nm, is very similar to what was reported for type 1, 5.0 nm (20), taking into account that a type 1 subunit is slightly larger. In that work, the length contribution of a single subunit was measured using a force-feedback approach. Thus, despite using diverse methods for measuring length changes of subunits that randomly shift between their bound and unbound state (force feedback or the stationary approach that we used), we end up with very similar values.

In addition to this, it is interesting to compare the force response of P and type 1 pili (both helical types) with those that are considered as linear fiber-like pili (23,44–46). Linear fiber-like pili differ both structurally and biomechanically from helical type pili. First, subunits assemble into a robust linear filament via covalent bonds, making the backbone strong against tensile force. Second, the lack of a helical shape results in a force response that is well modeled by a worm-like chain. Thus, these linear pili lack the force plateau of unwinding, and respond to tensile force in a manner similar to a stiff rope. This could be advantageous for bacteria that are exposed to very strong shear forces, such as when residing in the respiratory tract.

To conclude, our force measurement results indicate that the process of going from the bound to unbound state is faster than 10 μs, so we cannot resolve any intermediate steps. Therefore we cannot assess experimentally the detailed changes in subunit-subunit interactions that occur during uncoiling. However, a model from cryo-EM data shows multiple stabilizing interactions between subunits in the coiled configuration (9). Thus, we turned to MD simulations to investigate these interactions more closely and validate our results for the length change assessment upon uncoiling.

MD reveals mechanistic details of pilus uncoiling

We carried out sMD simulations of a 7mer segment of P pili. By simulating a 7mer filament, we could investigate the process of bond opening as the pilus was elongated in the direction of the filament axis. The sMD simulations were carried out at two different, constant pulling speeds, 5 Å/ns and 1 Å/ns, with five runs for each pulling speed. We also simulated a version of the 7mer system with the staple region removed; specifically, amino acids 1–5 were removed, because these are the amino acids that contact pilin subunits $n-4$ and $n-5$ from subunit n. These simulations were designed to investigate the contribution of staple residues to the total force required to extend the 7mer. Here we describe in detail the results of one run of the 1 Å/ns

simulations with the staple, which displayed very similar features compared with all other runs at both pulling speeds. Comparisons to the simulations carried out without the staple are described below. Details of the sMD protocol are described in the section "materials and methods," and data for all other runs of both systems are included in the supporting material.

To demonstrate the major reproducible features observed during filament extension, in Fig. 3 *A* we show all five runs of the 1 Å/ns sMD simulation force-extension curves plotted together. Each of the first four peaks corresponds to a particular subunit that was being pulled away from the 7mer filament. For example, peak I represents the subunit at the pilus tip (subunit 1) being pulled away from the filament, while peaks II–IV represent the subsequent breaking of layer-to-layer interactions for the second, third, and fourth subunits in the filament, respectively. Since we placed positional restraints on the bottom three subunits of the filament to provide a stable filament rod against which pulling occurred, we did not observe force peaks corresponding to extension of the bottom three subunits. Instead, because the base was restrained, as we continued to pull on the filament at constant speed, an irreversible breakage event occurred somewhere in the filament once the pilus was fully extended (e.g., a donated beta strand was pulled out from the groove it occupied in the n − 1 subunit). Peak V represents this breakage event, and the same behavior was observed in simulations of a P pilus 3mer (11). Force-extension curves for the additional simulations of the 7mer system at a pulling speed of 5 Å/ns, as well as the 7mer system without the staple at both pulling speeds, are included in supporting material Fig. S6. These data demonstrate that we observed similar features across both pulling speeds and across all simulation runs for the wild-type system. Finally, we note that filament breakage events occurred at various points along the filament in our simulations (e.g., the final panel in Fig. 3 *E*). Movies of each of the sMD trajectories are found in the supporting material (see Videos S1, S2, S3, and S4).

Specific features observed in the simulations are illustrated by a single run from the 1 Å/ns simulations in Fig. 3 *B*, *C*, *D*, and *E*. The lowercase letters at various points along the force-extension curve in Fig. 3 *B* correspond to the images in Fig. 3 *E* that depict representative snapshots from an sMD trajectory. The first peak (immediately after "a") and the peaks at "c," "e," and "g" correspond to bond-breaking events that occurred as subunits were pulled away from the filament rod. We note that these peaks coincide with the approximate extension at which the total number of contacts between subunits n and $n + 3$ (i.e., the subunit pairs that make lateral layer-to-layer interactions) began to fall rapidly toward zero (Fig. 3 *C*). Similarly, in Fig. 3 *B*, the valleys at "b," "d," "f," and "h" correspond to approximate extension lengths at which the contacts between a pair of n and $n + 3$ subunits had all been broken (Fig. 3 *C*); see supporting material Figs. S7–S10 for all plots of contacts versus filament extension, including systems both with and without the staple.

We observed that, at the point where the number of contacts reached zero between the n and $n + 3$ subunits, the angle of rotation of the newly freed subunit began to rapidly change with respect to the filament axis (Fig. 3 *D*, and Figs. S11–S14). We used the angle made by three amino acids (Gly 7, Val 18, and Cys 22), which are shown in the inset image of Fig. 3 *E*, as a proxy for the change in the angle of the subunit with respect to the filament axis. The angle between those amino acids started at approximately 95° and rotated to become nearly linear, corresponding to the linearization of the subunits as they were extended away from the filament rod due to the applied force (Fig. 3 *E*).

Taking Fig. 3 *B*, *C*, and *D* together as a representative example of filament extension, we can infer how the bond-breaking process generally occurred as the subunits were pulled away from the filament in the P pilus. For example, we can consider the points "b," "c," and "d" in Fig. 3 *B*, which demarcate a full force peak. At point "b," the force was beginning to increase to a maximum as the applied force was not yet strong enough to break all of the contacts between the second subunit (light gray) and the fifth subunit (orange) in the filament. Once the peak force was achieved at point "c," contacts began to rupture (Fig. 3 *C*, blue curve), and then, as the number of contacts dropped rapidly to zero, the force decreased between points "c" and "d." Once the force had reached a minimum at point "d," the second subunit was free to rotate, and became parallel to the filament axis (Fig. 3 *D*, blue curve). The force again began to increase between points "d" and "e," and the contacts between the next pair of n and $n + 3$ subunits began to rupture. This process was then repeated for each of the subunits in the filament. We therefore infer that, in a simulation of a much longer length of filament, we would continue to observe this sequential bond-breaking pattern, connecting the applied force, contact breakage, and subunit rotation.

Similar to the 4.3-nm bond-opening length observed in OT experiments, we note that the extension over which a subunit rotation occurred, as observed in Fig. 3 *D*, is also in this range. For example, for the pink curve (corresponding to the rotation of the terminal subunit), the rotation started at ~ 0 Å and stabilized at a nearly straight angle by ~ 35 Å extension. For the next three curves (blue, black, red), which correspond to rotation of the second (gray), third (goldenrod), and fourth (yellow) subunits respectively, the rotation from low angle to high angle occurred over approximately 45 Å, 40 Å, and 45 Å, respectively. Therefore, each bond-opening rotation of a subunit contributed ~ 35–45 Å of length toward extending the filament, in close agreement with the experimental data. Additional elongation outside of the bond-opening events is therefore related to additional

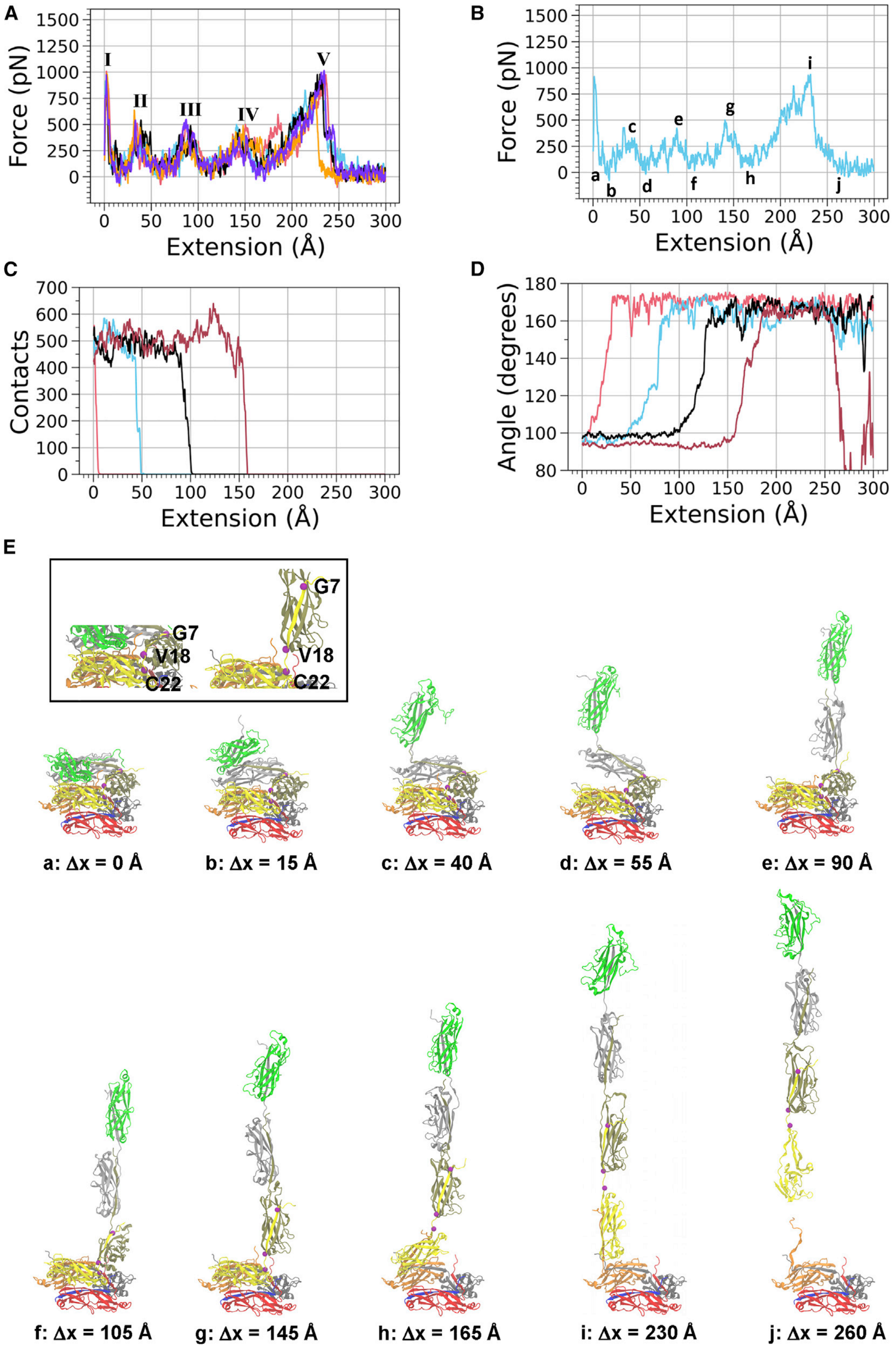

FIGURE 3 Force, contacts, and subunit interactions as P pili uncoil under force via sMD. (*A*) Force versus extension curves for all five of the v = 1 Å/ns sMD simulations with the staple present (pink = run 1, blue = run 2, black = run 3, orange = run 4, purple = run 5). Roman numerals I–IV identify the peak force at the point that a subunit was unwound from the filament, and Roman numeral V represents the force right as the 7mer was severed between two subunits (e.g., through the removal of a donated beta strand). The force curve data are a 1-ns running average. (*B*) The force versus extension data for

(legend continued on next page)

flexibility of other components of the subunits as, for example, was observed upon unfolding of an alpha helix within a subunit (11).

The staple region affects quaternary stability and subunit-subunit interactions

As discussed above, we were also interested in investigating the effects of the staple amino acids on the response of the P pilus to force. It has been previously reported that these amino acids contribute a significant amount of buried surface area to the interface between subunits in the pilus filament (9), and therefore these amino acids might provide additional stability against force. To ascertain the importance of the staple for pilus subunit-subunit interactions when pili are subjected to force, we deleted the first five amino acids at the N terminus of each subunit and repeated the sMD simulations.

For the simulations in which the staple was removed, we note that an overall lower average level of force was required to unwind each subunit from the helical filament, compared with simulations in which the staple was present (Fig 4 *A*, 1 Å/ns). This lower force for unwinding suggests that the staple amino acids provide stabilizing interactions to the pilus filament. The averaged force data for the systems both with and without the staple at a pulling speed of 5 Å/ns are shown in Fig. S6 *D*, which showed the same trend in the overall average level of force for the two systems as is observed in Fig. 4 *A*.

We also observed that, in simulations with the staple amino acids removed, contacts between subunits were eliminated over a smaller range of filament extension for the majority of runs, compared with simulations in which the staple was present (compare Figs. S7 and S8, as well as Figs. S9 and S10). To understand these trends in the contact breakage more fully, we also analyzed the contacts formed between the second subunit (light gray subunit in Fig. 3) with each of the four surrounding subunits (the fourth, fifth, sixth, and seventh subunits in the 7mer filament). The data for this analysis of the sMD simulations at 1 Å/ns are shown in Fig. 4 *B*, *C* (run 2), and all of the data for 1 Å/ns simulations are shown in Fig. S15. By analyzing the contacts separately instead of in aggregate, we can observe more directly how the interactions between a subunit being pulled off the filament rod and other subunits around it were disrupted. As expected, the largest number of contacts between subunits occurred for the layer-to-layer n and $n + 3$ interactions (orange curves). This was true for the simulations both with and without the staple. However, in simulations without the staple, the number of layer-to-layer contacts generally dropped more quickly than when the staple was present; with the staple present, the layer-to-layer contacts between subunits n and $n + 3$ began to drop roughly simultaneously with the contacts between the second and sixth subunits. Contacts formed between these two subunits primarily came from the staple region of the sixth subunit, as seen in the figures. We note that some interactions of the second subunit also occurred with the staple of the seventh subunit (red curves), and were therefore absent without the staple. Contacts between the second subunit and the fourth subunit were the last to be completely broken in each of the simulations, which occurred as the filament was straightened and pulled away from the filament rod (e.g., Fig. 3 *E*).

Finally, to define more specifically some of the important contacts that occur between amino acids in the P pilus, both in the presence and the absence of the staple amino acids, we analyzed data from our 100-ns equilibrium simulations to determine the most stable interactions. As seen in Tables S1–S3, we observed that some interactions found to be highly stable in the 100-ns equilibrium simulations were also noted as stabilizing residues in the P pilus structure by Hospenthal et al. (9). For example, a particularly large number of stable interactions to other amino acids was observed for Lys 125, Asp 126, and His 132 in our simulations. This finding is consistent with the importance of those amino acids for P pilus stability, as indicated by the mutation experiments in Hospenthal et al. demonstrating that their mutation led to diminished rod stability. Interestingly, in Hospenthal et al., the mutation of Thr 3 (slightly polar) to an arginine (positively charged) produced helical pili comparable with wild type, and, in our simulations without the staple, while we observed a decreased unwinding force for the P pilus, sMD elongation of the system is mechanistically similar to wild type. Together, the data suggest that Thr 3 is not a critical residue for pilus dynamics. Other changes that occurred when the staple was removed included a small number of new interactions that were only present in the equilibrium simulation without the staple (Table S4). It is possible that these new interactions could only occur after removal of steric hindrance by the staple, thereby producing a slightly stronger pilin-pilin interaction.

Mutation experiments in combination with OT force measurements, similar to the approach by Spaulding et al. (18),

run 2. Lowercase letters along the curve indicate which image in (*E*) of this figure corresponds to that degree of extension. (*C*) Total number of native plus non-native contacts between subunits 1 (tip subunit) and 4 (*pink curve*), 2 and 5 (*blue*), 3 and 6 (*black*), and 4 and 7 (*dark red*). For this analysis, a contact is defined as two atoms in a pair of subunits coming within a 4 Å cutoff distance of one another. The contact data are a 1-ns running average. (*D*) Angle between residues Gly 7, Val 18, and Cys 22 (*purple spheres* in *E*). The curves correspond to the alignment of subunit 1 (*pink curve*), subunit 2 (*blue curve*), subunit 3 (*black curve*) and subunit 4 (*dark red curve*) with the filament axis. When the angle is 90°, the subunit is perpendicular to the filament axis and, when the angle is 180°, the subunit is aligned with the filament axis. The angle data are a 1-ns running average. (*E*) Snapshots of the sMD simulation with letters that correspond to the labels in (*B*); purple spheres represent the positions of Gly 7, Val 18, and Cys 22. To see this figure in color, go online.

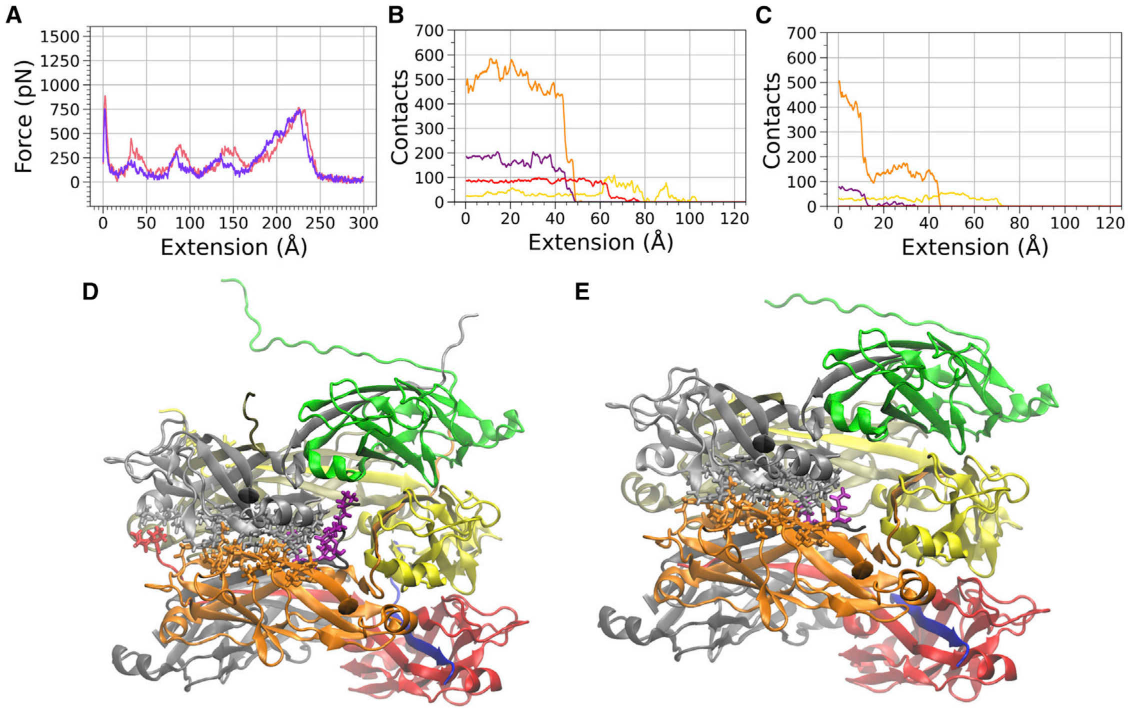

FIGURE 4 The staple region provides stability to P pili under force. (*A*) Force versus extension curves for the v = 1 Å/ns sMD simulations averaged over five separate runs for the system, including the staple (*pink*) and with the staple residues removed (*purple*); the data are a 1-ns running average. (*B*) Total number of contacts between subunits 2 and 4 (*yellow*), 2 and 5 (*orange*), 2 and 6 (*purple*), and 2 and 7 (*red*) for run 2. Contacts data are a 1-ns running average. They were calculated as described in Fig. 3 *C*. Data are for the simulations with the staple. (*C*) The same contact analysis as shown in (*B*), except for run 2 of the v = 1 Å/ns simulation *without* the staple. (*D* and *E*) Images of the 7mer with subunit 2 atoms (*light gray*) initially within 4 Å of subunits 4 (*yellow*), 5 (*orange*), 6 (*dark gray*), and 7 (*red*). The corresponding residues of subunits 4, 5, 6, and 7 that were within 4 Å of subunit 2 are also shown. For the residues on subunit 6, atoms are colored in purple for clarity. Colors of the lines in (*B*) and (*C*) are the same as the subunit coloring in (*D*) and (*E*), except the contacts between subunit 2 (*light gray*) and subunit 6 (*dark gray*) are also drawn in purple for clarity. To see this figure in color, go online.

could test the presence and role of a modified staple region in P pili. This would allow for an interesting comparison of P and type 1 pili since type 1 lacks the staple. It would then be possible to determine the contribution of the staple to the observed ~ 50-times higher bond-opening rate for P compared with type 1 pili (22), and explore a possible role for the staple in guiding subunits into their bound state during recoiling. This is plausible since the staple increases the reach of the layer-to-layer interactions, which could increase the probability of an unbound subunit finding its way back to the coiled subunits.

CONCLUSIONS

In conclusion, we investigated the biophysical properties of P pili and the structural interactions that stabilize this representative of the helical class of the CU adhesion pili family. Using high-resolution force-measuring OT, we unveil contour-length dependent compliance of helical pili: a fully helical pilus starts very stiff (700 pN/μm) but softens significantly during unwinding (100 pN/μm). We find that this biophysical property is well described by a coupled WLC and FJC model. Further, we assessed the kinetics and orientation change of subunits unbinding from the rod. We found that at steady state (the plateau force), at most six subunits are in the unbound state. When a single subunit switches from the bound state to the unbound state, the end-to-end length of the pilus increases 4.35 nm. A pilus can thus extend about six times its coiled length. Taken together, these experimental results imply that the donor strand does not restrict subunit movement during uncoiling, and acts as a hinge between the subunits, allowing the pilus to become fully linearized.

We verified these findings, investigated the bond-breaking process, and investigated the molecular interactions that stabilize the helical structure using 7mer filament sMD simulations. With a 7mer system, our simulations provide the first molecular-scale view of P pilus uncoiling at an atomistic level of detail, in a system large enough to include layer-to-layer interactions, aiding our interpretation of experimental force measurements. This detailed view of how interactions break and simulations in which the staple region was removed have provided valuable information about the role of the staple for P pilus stability. This study investigated essential biophysical and atomic features found in the helical class of uropathogenic adhesion pili and from our results we infer that the staple region significantly helps stabilize the helical rod structure.

SUPPORTING MATERIAL

Supporting material can be found online at https://doi.org/10.1016/j.bpj.2022.04.036.

AUTHOR CONTRIBUTIONS

T.D., J.B., E.B., and M.A. designed the research. T.D. and J.B. performed research. T.D., J.B., E.B., and M.A. analyzed data. M.A. was the project organizer. T.D., J.B., E.B., and M.A. wrote the manuscript. All authors read and agreed to the published version of the manuscript.

ACKNOWLEDGMENTS

This work was supported by the Swedish Research Council (2019-04016) to M.A. The authors acknowledge the facilities and technical assistance of the Umeå Core Facility for Electron Microscopy (UCEM) at the Chemical Biological Centre (KBC), Umeå University, a part of the National Microscopy Infrastructure NMI (VR-RFI 2016-00968). J.L.B. acknowledges support under NSF grant MCB-1817670. J.L.B. also acknowledges use of the Electronic Laboratory for Science and Analysis (ELSA) high-performance computing cluster at The College of New Jersey for conducting the simulations reported in this paper. This cluster was funded, in part, by the NSF under grants OAC-1826915 and OAC-1828163. This work was supported by the NIH, R21-AI-156236 to EB.

DECLARATION OF INTERESTS

The authors declare no competing interests.

REFERENCES

1. Thanassi, D. G., E. T. Saulino, and S. J. Hultgren. 1998. The chaperone/usher pathway: a major terminal branch of the general secretory pathway. *Curr. Opin. Microbiol.* 1:223–231. https://doi.org/10.1016/s1369-5274(98)80015-5.

2. Leffler, H., and C. S. Edén. 1980. Chemical identification of a glycosphingolipid receptor for Escherichia coli attaching to human urinary tract epithelial cells and agglutinating human erythrocytes. *FEMS Microbiol. Lett.* 8:127–134. https://doi.org/10.1111/j.1574-6968.1980.tb05064.x.

3. Lane, M. C., and H. L. T. Mobley. 2007. Role of P-fimbrial-mediated adherence in pyelonephritis and persistence of uropathogenic Escherichia coli (UPEC) in the mammalian kidney. *Kidney Int.* 72:19–25. https://doi.org/10.1038/sj.ki.5002230.

4. Sauer, F. G., H. Remaut, ..., G. Waksman. 2004. Fiber assembly by the chaperone-usher pathway. *Biochim. Biophys. Acta.* 1694:259–267. https://doi.org/10.1016/j.bbamcr.2004.02.010.

5. Roberts, J. A., B. I. Marklund, ..., S. Normark. 1994. The Gal(alpha 1-4)Gal-specific tip adhesin of Escherichia coli P-fimbriae is needed for pyelonephritis to occur in the normal urinary tract. *Proc. Natl. Acad. Sci. U S A.* 91:11889–11893. https://doi.org/10.1073/pnas.91.25.11889.

6. Jones, C. 1997. The chaperone-assisted membrane release and folding pathway is sensed by two signal transduction systems. *EMBO J.* 16:6394–6406. https://doi.org/10.1093/emboj/16.21.6394.

7. Choudhury, D., A. Thompson, ..., S. D. Knight. 1999. X-ray structure of the FimC-FimH chaperone-adhesin complex from uropathogenic Escherichia coli. *Science.* 285:1061–1066. https://doi.org/10.1126/science.285.5430.1061.

8. Bullitt, E., and L. Makowski. 1995. Structural polymorphism of bacterial adhesion pili. *Nature.* 373:164–167. https://doi.org/10.1038/373164a0.

9. Hospenthal, M., A. Redzej, ..., G. Waksman. 2016. Structure of a chaperone-usher pilus reveals the molecular basis of rod uncoiling. *Cell.* 164:269–278. https://doi.org/10.1016/j.cell.2015.11.049.

10. Zheng, W., M. Andersson, ..., E. Egelman. 2019. Cryo-EM structure of the CFA/I pilus rod. *IUCrJ.* 6:815–821. https://doi.org/10.1107/s2052252519007966.

11. Baker, J. L., T. Dahlberg, ..., M. Andersson. 2021. Impact of an alpha helix and a cysteine – cysteine disulfide bond on the resistance of bacterial adhesion pili to stress. *Proc. Natl. Acad. Sci. U S A.* 118. . e2023595118. https://doi.org/10.1073/pnas.2023595118.

12. Andersson, M., O. Björnham, ..., E. Bullitt. 2012. A structural basis for sustained bacterial adhesion: biomechanical properties of CFA/I pili. *J. Mol. Biol.* 415:918–928. https://doi.org/10.1016/j.jmb.2011.12.006.

13. Thomas, W. E., E. Trintchina, ..., E. V. Sokurenko. 2002. Bacterial adhesion to target cells enhanced by shear force. *Cell.* 109:913–923. https://doi.org/10.1016/s0092-8674(02)00796-1.

14. Sokurenko, E. V., V. Vogel, and W. E. Thomas. 2008. Catch-bond mechanism of force-enhanced adhesion: counterintuitive, elusive, but, widespread? *Cell Host and Microbe.* 4:314–323. https://doi.org/10.1016/j.chom.2008.09.005.

15. Rangel, D. E., N. Marín-Medina, ..., M. Forero-Shelton. 2013. Observation of bacterial type I pili extension and contraction under fluid flow. *PLoS One.* 8:e65563. https://doi.org/10.1371/journal.pone.0065563.

16. Zakrisson, J., K. Wiklund, ..., M. Andersson. 2012. Helix-like biopolymers can act as dampers of force for bacteria in flows. *Eur. Biophys. J.* 41:551–560. https://doi.org/10.1007/s00249-012-0814-8.

17. Zakrisson, J., K. Wiklund, ..., M. Andersson. 2015. Tethered cells in fluid flows – beyond the Stokes' drag force approach. *Phys. Biol.* 12:056006. https://doi.org/10.1088/1478-3975/12/5/056006.

18. Spaulding, C. N., H. L. Schreiber, ..., E. H. Egelman. 2018. Functional role of the type 1 pilus rod structure in mediating host-pathogen interactions. *eLife.* 7:e31662. https://doi.org/10.7554/elife.31662.

19. Jass, J., S. Schedin, ..., O. Axner. 2004. Physical properties of Escherichia coli P pili measured by optical tweezers. *Biophysical J.* 87:4271–4283. https://doi.org/10.1529/biophysj.104.044867.

20. Miller, E., T. Garcia, ..., A. F. Oberhauser. 2006. The mechanical properties of E. coli type 1 pili measured by atomic force microscopy techniques. *Biophysical J.* 91:3848–3856. https://doi.org/10.1529/biophysj.106.088989.

21. Andersson, M., E. Fällman, ..., O. Axner. 2006. A sticky chain model of the elongation and unfolding of Escherichia coli P pili under stress. *Biophysical J.* 90:1521–1534. https://doi.org/10.1529/biophysj.105.074674.

22. Andersson, M., B. E. Uhlin, and E. Fällman. 2007. The biomechanical properties of E. coli pili for urinary tract attachment reflect the host environment. *Biophysical J.* 93:3008–3014. https://doi.org/10.1529/biophysj.107.110643.

23. Castelain, M., E. Koutris, ..., O. Axner. 2009. Characterization of the biomechanical properties of T4 pili expressed by Streptococcus pneumoniae–a comparison between helix-like and open coil-like pili. *ChemPhysChem.* 10:1533–1540. https://doi.org/10.1002/cphc.200900195.

24. Castelain, M., S. Ehlers, ..., O. Axner. 2011. Fast uncoiling kinetics of F1C pili expressed by uropathogenic Escherichia coli are revealed on a single pilus level using force-measuring optical tweezers. *Eur. Biophys. J.* 40:305–316. https://doi.org/10.1007/s00249-010-0648-1.

25. Chen, F.-J., C.-H. Chan, ..., L. Hsu. 2011. Structural and mechanical properties of Klebsiella pneumoniae type 3 fimbriae. *J. Bacteriol.* 193:1718–1725. https://doi.org/10.1128/jb.01395-10.

26. Mortezaei, N., C. R. Epler, ..., E. Bullitt. 2015a. Structure and function of enterotoxigenic Escherichia coli fimbriae from differing assembly pathways. *Mol. Microbiol.* 95:116–126. https://doi.org/10.1111/mmi.12847.

27. Mortezaei, N., B. Singh, …, M. Andersson. 2015b. Biomechanical and structural features of CS2 fimbriae of enterotoxigenic Escherichia coli. *Biophysical J.* 109:49–56. https://doi.org/10.1016/j.bpj.2015.05.022.

28. Båga, M., M. Göransson, …, B. E. Uhlin. 1988. Processed mRNA with differential stability in the regulation of E. coli pilin gene expression. *Cell.* 52:197–206. https://doi.org/10.1016/0092-8674(88)90508-9.

29. Bergsten, G., B. Wullt, and C. Svanborg. 2005. Escherichia coli, fimbriae, bacterial persistence and host response induction in the human urinary tract. *Int. J. Med. Microbiol.* 295:487–502. https://doi.org/10.1016/j.ijmm.2005.07.008.

30. Lindberg, S., Y. Xia, …, B. E. Uhlin. 2008. Regulatory Interactions among adhesin gene systems of uropathogenic Escherichia coli. *Infect. Immun.* 76:771–780. https://doi.org/10.1128/iai.01010-07.

31. Stangner, T., T. Dahlberg, …, M. Andersson. 2018. Cooke – triplet tweezers: more compact, robust, and efficient optical tweezers. *Opt. Lett.* 43:1990. https://doi.org/10.1364/ol.43.001990.

32. Andersson, M., F. Czerwinski, and L. B. Oddershede. 2011. Optimizing active and passive calibration of optical tweezers. *J. Opt.* 13:044020. https://doi.org/10.1088/2040-8978/13/4/044020.

33. Tolic-Norrelykke, S. F., E. Schaffer, …, H. Flyvbjerg. 2006. Calibration of optical tweezers with positional detection in the back focal plane. *Rev. Scientific Instr.* 77:103101. https://doi.org/10.1063/1.2356852.

34. Case, D., H. Aktulga, …, P. Kollman. 2021. Amber 2021, University of California. https://ambermd.org/.

35. Lugmaier, R. A., S. Schedin, …, M. Benoit. 2008. Dynamic restacking of Escherichia coli P-pili. *Eur. Biophys. J.* 37:111–120. https://doi.org/10.1007/s00249-007-0183-x.

36. Björnham, O., O. Axner, and M. Andersson. 2008. Modeling of the elongation and retraction of Escherichia coli P pili under strain by Monte Carlo simulations. *Eur. Biophys. J.* 37:381–391. https://doi.org/10.1007/s00249-007-0223-6.

37. Mu, X.-Q., and E. Bullitt. 2006. Structure and assembly of P-pili: a protruding hinge region used for assembly of a bacterial adhesion filament. *Proc. Natl. Acad. Sci. U S A.* 103:9861–9866. https://doi.org/10.1073/pnas.0509620103.

38. Bullitt, E., and L. Makowski. 1998. Bacterial adhesion pili are heterologous assemblies of similar subunits. *Biophysical J.* 74:623–632. https://doi.org/10.1016/s0006-3495(98)77821-x.

39. Andersson, M., E. Fällman, O. Axner…, 2006. Technique for determination of the number of PapA units in an E. Coli P pilus. *Proc. SPIE* 608814. https://doi.org/10.1117/12.642261.

40. Andersson, M., O. Axner, …, E. Fällman. 2008. Physical properties of biopolymers assessed by optical tweezers: analysis of folding and refolding of bacterial pili. *ChemPhysChem.* 9:221–235. https://doi.org/10.1002/cphc.200700389.

41. Castelain, M., A. E. Sjöström, …, M. Andersson. 2010. Unfolding and refolding properties of S pili on extraintestinal pathogenic Escherichia coli. *Eur. Biophys. J.* 39:1105–1115. https://doi.org/10.1007/s00249-009-0552-8.

42. Habenstein, B., A. Loquet, A. Lange…, 2015. Hybrid structure of the type 1 pilus of uropathogenic Escherichia coli. *Angew. Chem.* 127:11857–11861. https://doi.org/10.1002/ange.201505065.

43. Hospenthal, M. K., D. Zyla, …, G. Waksman. 2017. The cryoelectron microscopy structure of the type 1 chaperone-usher pilus rod. *Structure.* 25:1829–1838.e4. https://doi.org/10.1016/j.str.2017.10.004.

44. Tripathi, P., A. Beaussart, …, Y. F. Dufrêne. 2013. Adhesion and nanomechanics of pili from the probiotic Lactobacillus rhamnosus GG. *ACS Nano.* 7:3685–3697. https://doi.org/10.1021/nn400705u.

45. Castelain, M., M.-P. Duviau, …, M. Mercier-Bonin. 2016. The nanomechanical properties of Lactococcus lactis pili are conditioned by the polymerized backbone pilin. *PLoS One.* 11:e0152053. https://doi.org/10.1371/journal.pone.0152053.

46. Mignolet, J., A. Viljoen, …, Y. F. Dufrêne. 2021. AFM unravels the unique adhesion properties of the Caulobacter type IVc pilus nanomachine. *Nano Lett.* 21:3075–3082. https://doi.org/10.1021/acs.nanolett.1c00215.

Biophysical Journal, Volume *121*

Supplemental information

Unveiling molecular interactions that stabilize bacterial adhesion pili

Tobias Dahlberg, Joseph L. Baker, Esther Bullitt, and Magnus Andersson

Unveiling molecular interactions that stabilize bacterial adhesion pili

Tobias Dahlberg[a,e], Joseph L. Baker[b,e], Esther Bullitt[c,*], and Magnus Andersson[a,d,*]

[a]Department of Physics, Umeå University, Umeå, 90187, Sweden;
[b]Department of Chemistry, The College of New Jersey, Ewing, NJ, 08628, USA;
[c]Department of Physiology & Biophysics, [c]Boston University School of Medicine, Boston, MA, 02118, USA
[d]Umeå Centre for Microbial Research (UCMR), Umeå, Sweden
[e]Contributed equal to the work

[*]Corresponding authors: bullitt@bu.edu and magnus.andersson@umu.se

Supporting Material content

S0 Optical tweezers instrumentation and sample preparation 2

S1 Supporting force spectroscopy information and pilus modeling 5
Measuring stiffness of an uncoiled pilus 5
Compliance correction 6
Stiffness model for an uncoiling pilus 6

S2 Supporting molecular dynamics information 10
Contact analysis in the 100 ns equilibrium simulations 20

S3 Detailed molecular dynamics simulation methods 24
Preparation for steered molecular dynamics (sMD) simulations 24
sMD simulations 26
Extended equilibrium simulations 27
Steered Molecular Dynamics Movie Captions 27

References 28

S0 Optical tweezers instrumentation and sample preparation

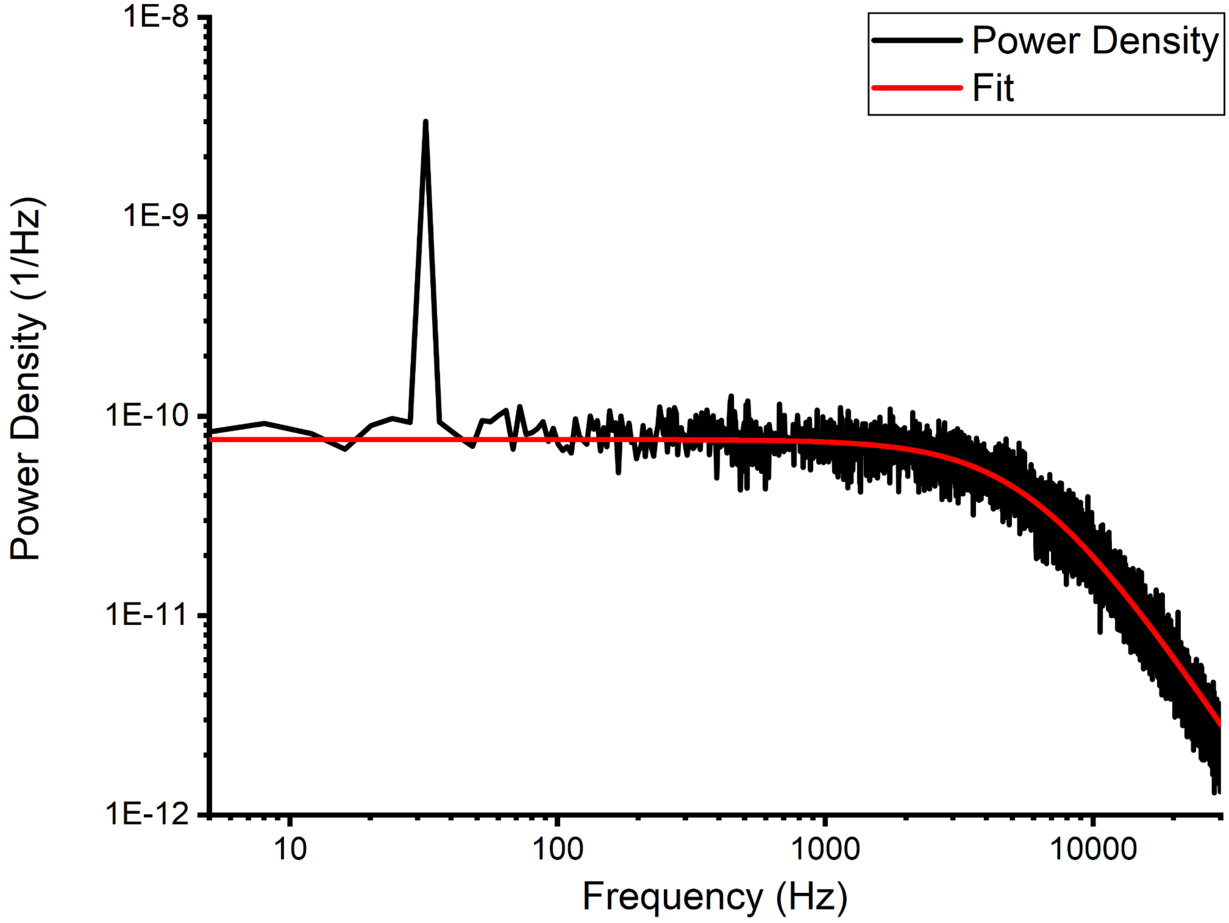

Figure S1. Example of an experimental power spectrum of a trapped bead (black). We have fitted the power spectrum with a Lorenztian model (red) given by $y = A/(f^2 + f_c^2)$ where f is the frequency, f_c the corner frequency and A a fitting parameter related to the diffusion coefficient of the trapped bead and sensitivity of the detection system. The resulting fitting parameters are $A = 0.002663$, and $f_c = 5907$ Hz. The trapped bead is oscillated at 32 Hz, whose response is clearly visible in the data as a sharp peak in the power spectrum.

The optical tweezers instrumentation stands in a temperature controlled room on an actively isolated optical table (TMC). To reduce electrical and acoustic noise in the setup, we place computers and controllers in a separate room. We form the trap using a 1064 nm DPSS laser (Cobolt Rumba, Cobolt AB, Solna, Sweden) with a maximum power of 2 W. The laser beam is expanded to fill the numerical aperture of our microscope objective and introduced into the microscope using a dichroic mirror (DMSP650, Thorlabs, Newton, NJ). The laser light is then transmitted by the sample, scattered by the trapped object, and collected by the condenser. The collected light then interferes in the back focal plane of the condenser, which we image onto a 2D position-sensitive detector (PSD, 2L10YAG SU65 SPC02, Sitek Electro Optics, Sweden). The PSD tracks the centroid of the interference pattern and converts it to a voltage. We then

filter the voltage with a programmable low pass anti-aliasing filter (LTC1064-2, Analog Devices, Wilmington, MA) before measuring it using a computer equipped with a data acquisition (DAQ) card. We then process the collected data using LabVIEW.

To prepare a sample, we first suspend bacteria in 1xPBS to a concentration of 1:1000 from OD_{600} = 1. Then we also suspend 1.04 μm polystyrene microspheres (4010A, Thermo Fisher Scientific, Waltham, MA) in 1xPBS. These microspheres are later used as force probes. To create a stable mounting point for bacteria, we prepare a 1:500 suspension of 9.5 um carboxylate-modified latex microspheres (product no. 2-10000, Interfacial Dynamics, Portland, OR) in Milli-Q water. 10 μl of this bead suspension is then dropped on a 24 x 60 mm coverslip (no. 1, Paul Marienfeld GmbH, Lauda-Königshofen, Germany). To fix these beads on the surface of the coverslip, we dry them in an oven for 60 min at 60 C. Then, we add a solution of 20 μl of 0.01 % poly-L-lysine (catalog no. P4832, Sigma-Aldrich, St. Louis, MO) to the coverslip, which we dry for 45 min at 37 C. This layer of poly-L-lysine helps the bacteria stay firmly attached during the measurement process.

To construct a sample chamber, we first add two strips of double-sided tape (Scotch, product no. 34-8509-3289-7, 3M) to one of the bead-coated coverslips. Then we place a 20 x 20 mm coverslip (no.1, Paul Marienfeld GmbH, Lauda-Königshofen, Germany) on top of the tape. This process forms a small gap between the lower and upper coverslips, and the tape acts as a spacer and a sealant. After that, we let capillary forces draw in 2 μl of suspended bacteria and 10 μl of suspended beads through the open sides of the chamber. We then seal these open sides using vacuum grease (Dow Corning, Midland, MI). Finally, we mount the completed sample chamber in a sample holder fixed to a piezo-stage (Physik Instrument, P-561.3CD stage) in the OT instrumentation.

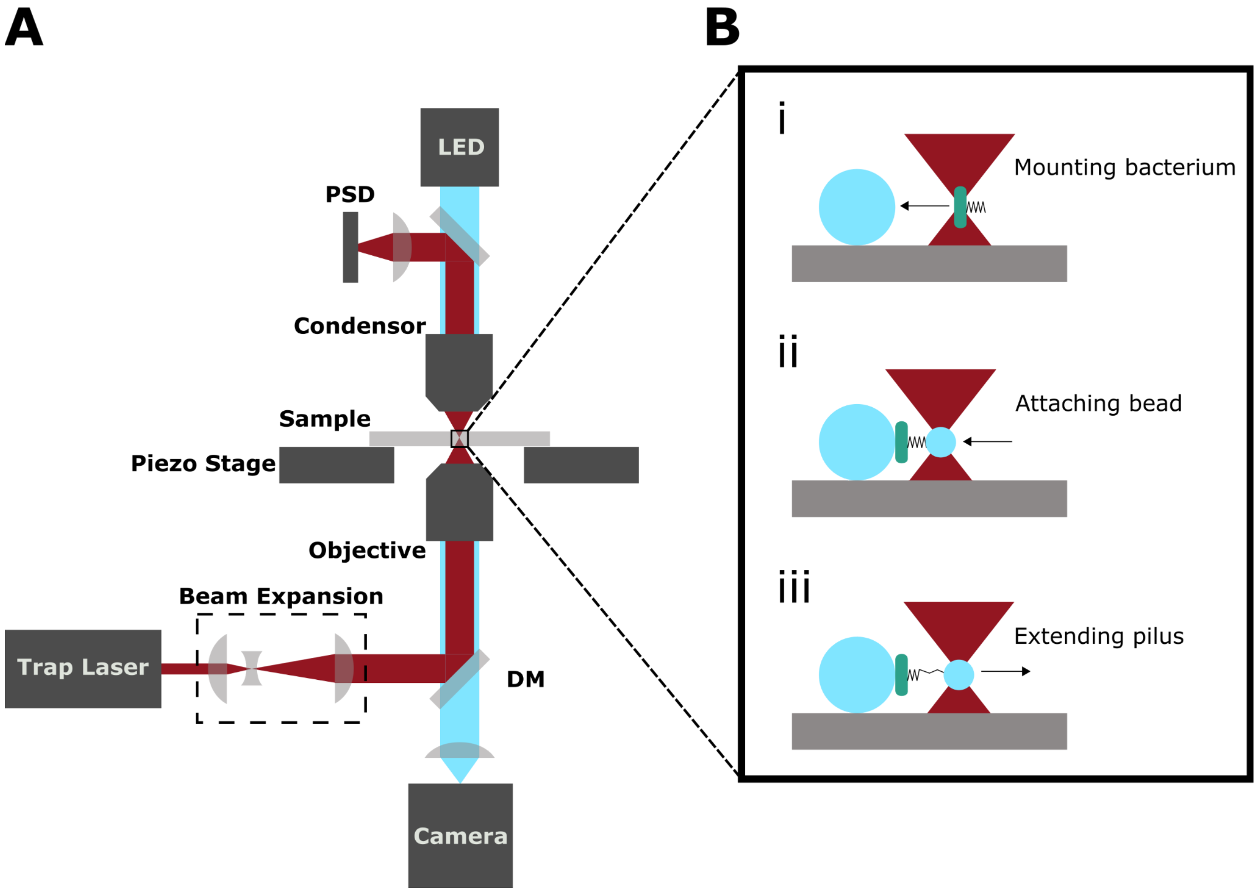

Figure S2. Schematic of the optical tweezer setup used to measure the coiling and uncoiling of pili. (A) We introduce the trap laser beam into the microscope using a dichroic mirror (DM). The laser beam is then focused by the objective into the sample to form the trap. To measure the position of a trapped microsphere we collect the light scattered by the sphere and transmitted through the sample with the condenser. The collected light interferes in the back focal plane of the condenser which we image on a 2D PSD. The PSD measures the centroid of the interference pattern and converts it to a voltage that we measure using a computer. (B) An illustration of a force-extension measurement. i) We trap a single bacterium and mount it onto a large microsphere that is fixed to the bottom coverslip of the sample chamber. ii) We trap a small microsphere that we attach to a pilus on the bacterium. iii) We separate the bacterium and trapped microsphere to apply a tensile force to the pilus and extend it partially. Then we track how the coiling and uncoiling of the pilus displaces the bead in the trap versus time.

S1 Supporting force spectroscopy information and pilus modeling

Measuring stiffness of an uncoiled pilus

Due to the inherent force clamping of an unwinding pilus, we cannot easily assess its stiffness directly. Therefore, to measure the stiffness of a partially unwound pilus, we use the equipartition theorem. This method allows us to assess the stiffness of the harmonic potential formed by both the trap and the pilus. For small displacements, as in our setup, we can assume that both the trap and the pilus are harmonic. Thus, we can estimate the total stiffness of trap and pilus, k_{tot}, using the equipartition theorem defined as,

$$k_{tot} = \frac{k_B T}{<x^2>}, \quad (1)$$

where k_B is Boltzmann's constant, T is the temperature, and $<x^2>$ is the variance of the trapped bead's position. As the pilus and optical trap are connected to the bead in parallel, their stiffnesses should be additive [1]. Thus, we can estimate the stiffness of the pilus, k_p, using our measured value for k_{tot} and k_t using

$$k_p = k_{tot} - k_t. \quad (2)$$

To estimate the stiffness of a pilus from our data, we used a moving variance with a window length of 3000 samples. We converted this moving variance to stiffness using Eq. 1 and 2 and created a histogram of the values. From this histogram, we estimated the most likely stiffness by taking the stiffness with the most counts. We show an example of two histograms like this for a pilus extended 3 µm and 8 µm into region II, Figure S3. This figure shows that the measured stiffness decreases as the pilus gets extended, and the histogram approaches a normal distribution. The asymmetry of the stiffer grey histogram comes from the contribution of the binding and unbinding events, which create high variance spikes in the signal. This effect disappears for the less stiff red histogram because when the stiffness goes down, we lose temporal and spatial resolution and do not resolve these binding and unbinding events clearly.

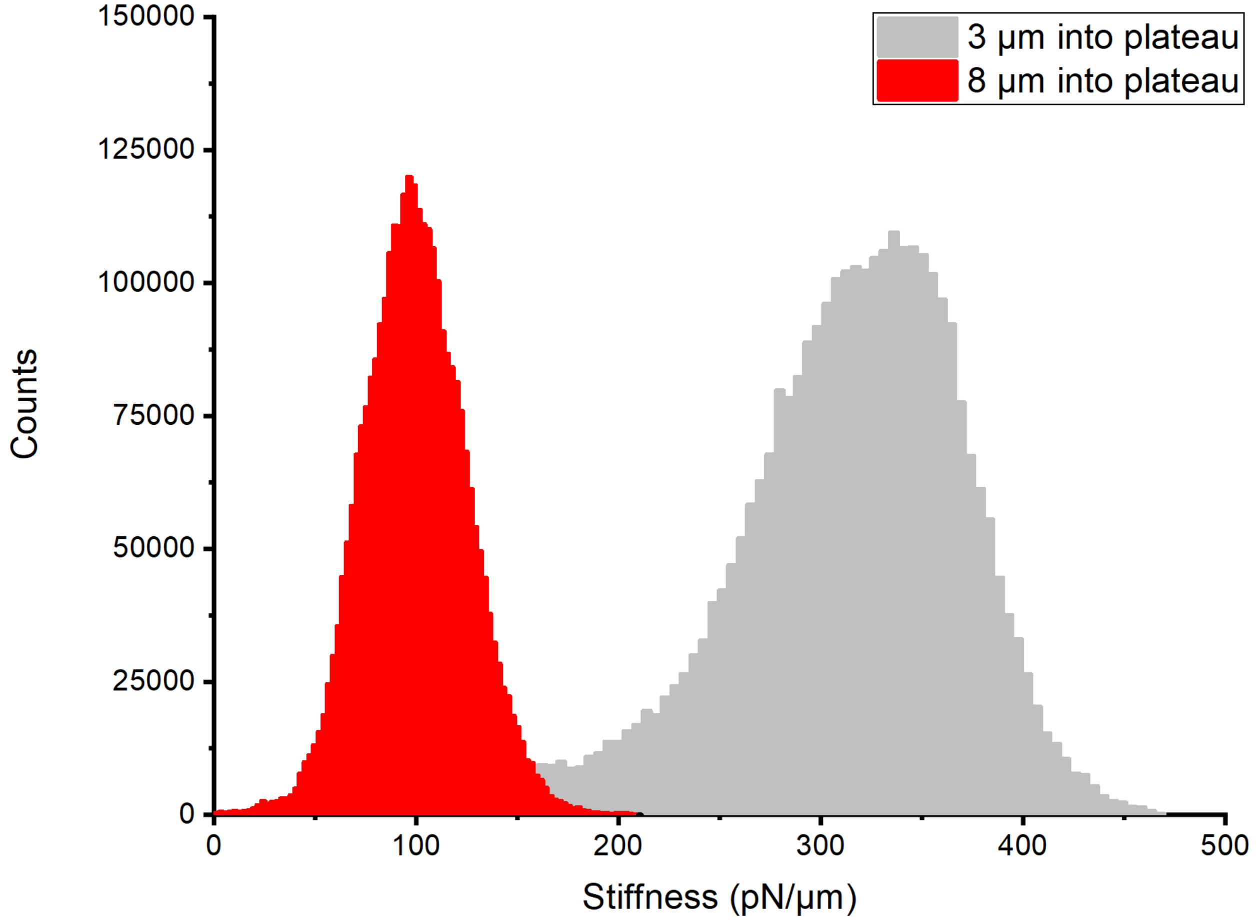

Figure S3. Stiffness of pilus measured using a moving variance histogram of the force data with a window length of 3000 samples for two different extensions of the same pilus.

Compliance correction

When measuring the length changes of a pilus when a subunit loses and regains contact with its neighbors using an optical tweezer, in which the trap's position is held constant, we need to account for the fact that the force applied to the pilus does not stay constant as it shortens or elongates. This changing force will change the relative degree of extension of the pilus at the same time as it elongates or shortens. This effect causes our measured displacement of the bead to be less than the actual length change of the pilus [1]. The relationship between the displacement of the bead, x_m, and the length change of the pilus, ΔR , is given by

$$x_m = \Delta R \frac{k_t + k_p}{k_p}. \tag{3}$$

Stiffness model for an uncoiling pilus

We model an uncoiling pilus as a combination of an extensible worm like chain (WLC) [2], representing the coiled rod, and a freely jointed chain (FJC) [3], representing the uncoiled linear

region. For the uncoiled region the FJC end-to-end distance, R_u, versus force, F, relationship is given by

$$R_u = L_u(coth(\frac{FL_k}{k_BT}) - \frac{k_BT}{Fl_k}), \tag{5}$$

where T is the temperature, k_B is Boltzmann's constant, L_u is the contour length, and l_k is the Kuhn length. Here, the end-to-end distance R_u is the straight line between the first and last subunit, whereas the contour length L_u is the length of the backbone of all subunits in the uncoiled configuration, as diagrammed in Figure S4.

For the coiled region, the WLC end-to-end distance, R_c, versus force relationship is given by

$$R_c = L_c(1 - \frac{1}{2}(\frac{k_BT}{FP_c})^{1/2} + \frac{F}{K_c}), \tag{4}$$

where, L_c is the contour length, P_c is the persistence length, and K_c is the enthalpic stiffness of the coiled pilus. Thus, the total end-to-end distance R_{tot} vs force for the pilus, if we assume that the coiled and uncoiled regions are experiencing the same force, is given by

$$R_{tot} = R_u + R_c. \tag{6}$$

To model the uncoiling of the pilus we treat it as being composed of N_{tot} subunits where N_b is the number of subunits in the bound state that forms the coiled region of the pilus and N_u is the number in the unbound state, forming the uncoiled region, and $N_{tot} = N_c + N_u$. Each subunit in these two states contributes to the total contour length of the pilus with the lengths x_b and x_u for the bound and unbound states, respectively. Thus, the total contour length can be calculated by

$$L_{tot} = L_u + L_c = N_b x_b + N_u x_u. \tag{7}$$

During uncoiling, the pilus will start with all subunits in the bound state, so N_b=N_{tot}, and transition to a fully uncoiled structure with N_u=N_{tot}. This uncoiling will change the contour lengths of the coiled and uncoiled regions of the structure, leading to an end-to-end distance versus force relationship that changes with the degree of uncoiling. By combining these equations, we can express the total end-to-end distance versus force as a function of the number of unbound subunits in the following way

$$R_{tot}(F, N_u) = N_u x_u(coth(\frac{Fl_k}{k_B T}) - \frac{k_B T}{Fl_k}) + (N_{tot} - N_u)x_c(1 - \frac{1}{2}(\frac{k_B T}{FP_c})^{1/2} + \frac{F}{K_c}). \quad (8)$$

From this equation, we can relate the change in end-to-end distance, ΔR_{tot}, when a subunit unbinds, to the actual change in total contour length, ΔL_{tot}, by the following equations

$$\Delta R_{tot} = R_{tot}(F, N_u) - R_{tot}(F, N_u + 1) = x_b(1 - \frac{1}{2}(\frac{k_B T}{Fc})^{1/2} + \frac{F}{K_c}) - x_u(coth(\frac{Fl_k}{k_B T}) - \frac{k_B T}{Fl_k}), \quad (9)$$

$$\Delta L_{tot} = x_u - x_b. \quad (10)$$

Further, by taking the inverse of $\frac{dR_{tot}(F, N_u)}{dF}$ we can get the stiffness, k_{pili}, of a partially uncoiled pilus as

$$k_{pili} = (\frac{dR_{tot}(F, N_u)}{dF})^{-1} = (x_u N_u(\frac{k_b T}{F^2 l_k} - csch^2(\frac{Fl_k}{k_b T})) + x_b(N_{tot} - N_u)(\frac{1}{4}(\frac{k_b T}{FP_c})^{-1/2}\frac{k_b T}{F^2 P_c} + \frac{1}{K_c}))^{-1}. \quad (11)$$

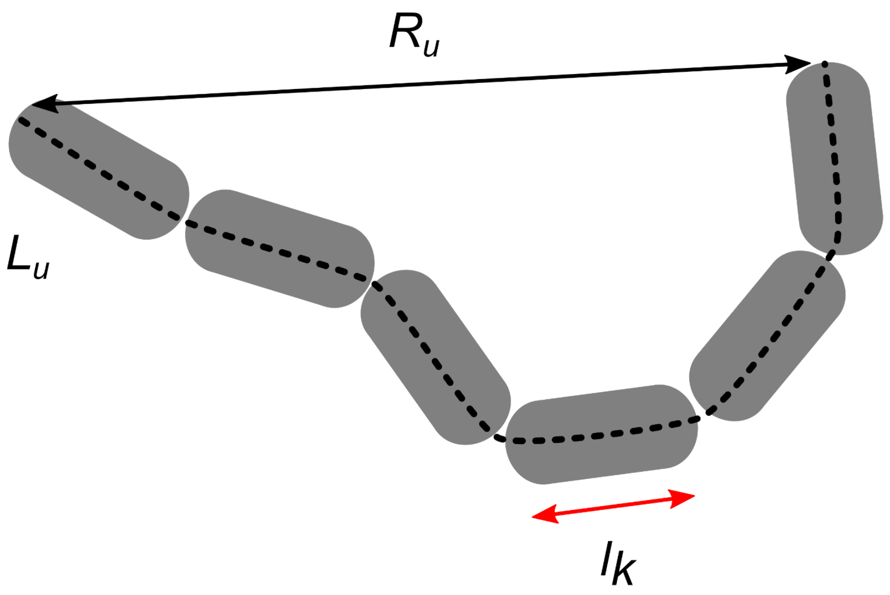

Figure S4 Schematic showing the difference between end-to-end distance R_u(solid line), and contour length L_u(dashed line) of a polymer assembled from subunits with a Kuhn length l_k(red line).

To validate this model, we measured the stiffness of P pili (measure on 3 individual pili) for various degrees of uncoiling using the method outlined below. Comparing these data to the calculated stiffness using our model with parameters P_c = 1 μm, K_c = 1300 pN, N_{tot} = 2000, l_k = 5.1 nm, x_b = 0.754 nm, F = 28 pN, and x_u = 5.1 nm, shows good agreement, Figure S5. As the value l_k represents the length of a link in the FJC, the fact that our result closely matches the length of a PapA subunit, ~5 nm, lends credence to our model. Further, if we compare this to a previously used stiffness model for P pili [4], we see that this older model did not reflect our measured stiffness versus elongation behavior and underestimated the pilus stiffness. This indicates that it is more suitable to model the uncoiling pilus as a WLC connected in series with a FJC.

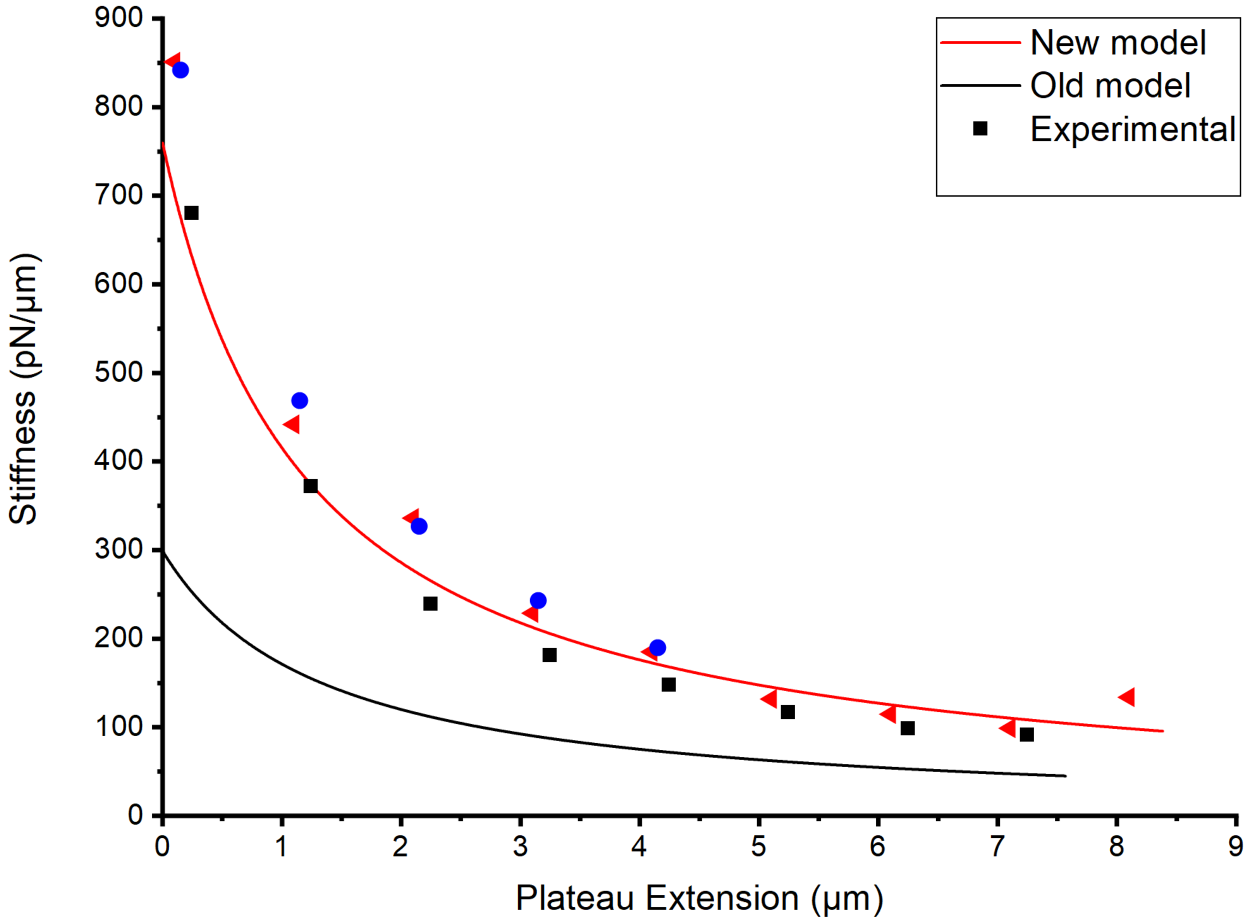

Figure S5. A comparison of our stiffness model (red line), previous stiffness model (black line), and three experimental data sets represented by the red triangles, blue dots and black squares.

S2 Supporting molecular dynamics information

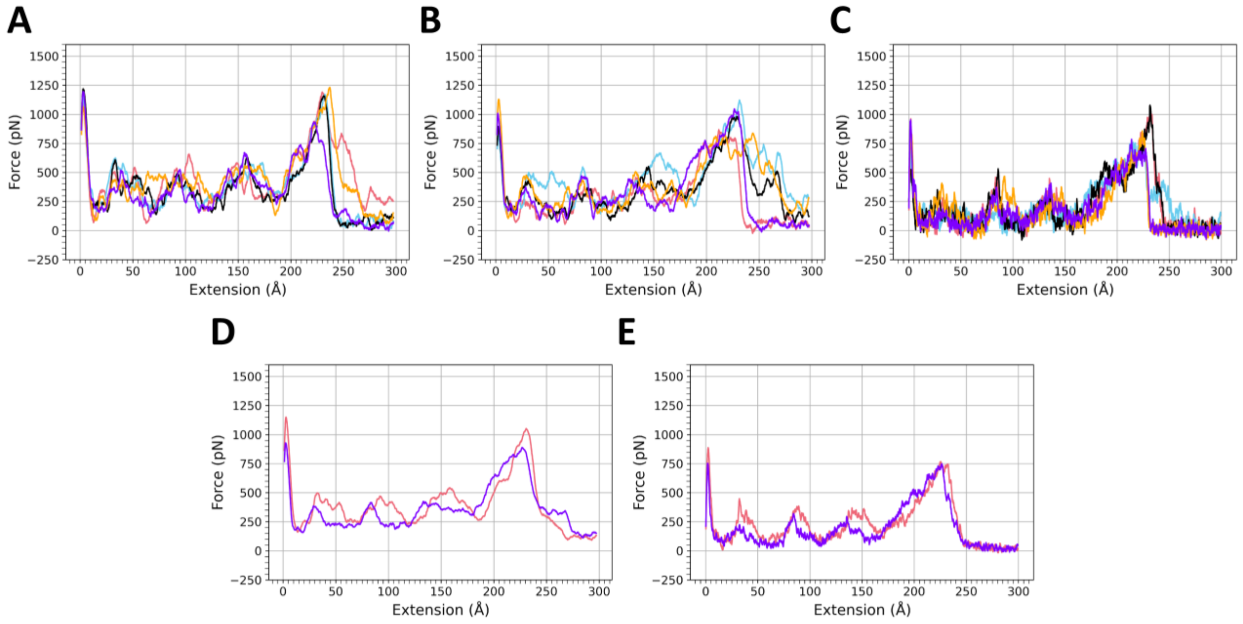

Figure S6. Force versus extension curves for all five of the v = 5 Å/ns steered molecular dynamics simulations with (A) the staple residues present, and (B) the staple residues removed. (C) Force versus extension curves for all five of the v = 1 Å/ns steered molecular dynamics simulations with the staple residues removed. The corresponding figure with the staple residues present for the v = 1 Å/ns steered molecular dynamics simulations is Figure 3 of the main text. In panels (A), (B) and (C) pink = run 1, blue = run 2, black = run 3, orange = run 4, and purple = run 5. (D) Force versus extension curves for the v = 5 Å/ns steered molecular dynamics simulations averaged over the five separate runs for the system including the staple (pink) and with the staple residues removed (purple). (E) Force versus extension curves for the v = 1 Å/ns steered molecular dynamics simulations averaged over the five separate runs for the system including the staple (pink) and with the staple residues removed (purple). The force curve data are a 1 ns running average.

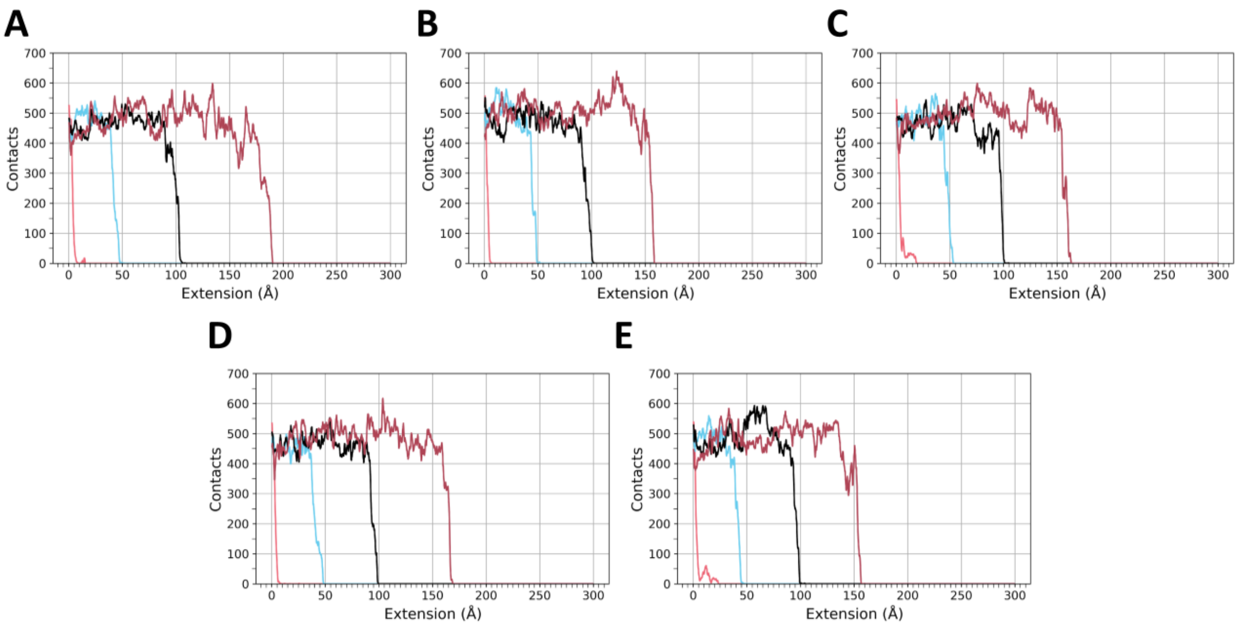

Figure S7. Total number of contacts between subunits 1 and 4 (pink), 2 and 5 (blue), 3 and 6 (black), and 4 and 7 (dark red), for the v = 1 Å/ns simulations (A) run 1, (B) run 2, (C) run 3, (D) run 4, and (E) run 5. Data are plotted as a function of extension for ease of comparison to the force vs extension curves. The contacts data are a 1 ns running average. Data are for the simulations with the staple.

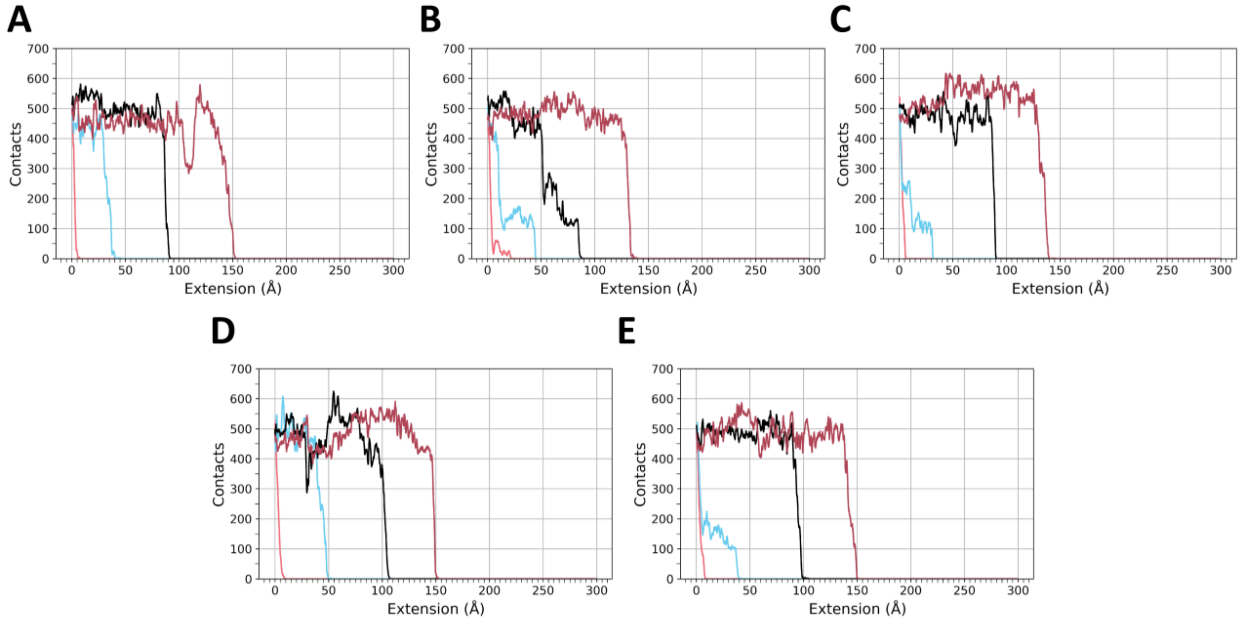

Figure S8. Total number of contacts between subunits 1 and 4 (pink), 2 and 5 (blue), 3 and 6 (black), and 4 and 7 (dark red), for the v = 1 Å/ns simulations (A) run 1, (B) run 2, (C) run 3, (D) run 4, and (E) run 5. Data are plotted as a function of extension for ease of comparison to the force vs extension curves. The contacts data are a 1 ns running average. Data are for the simulations without the staple.

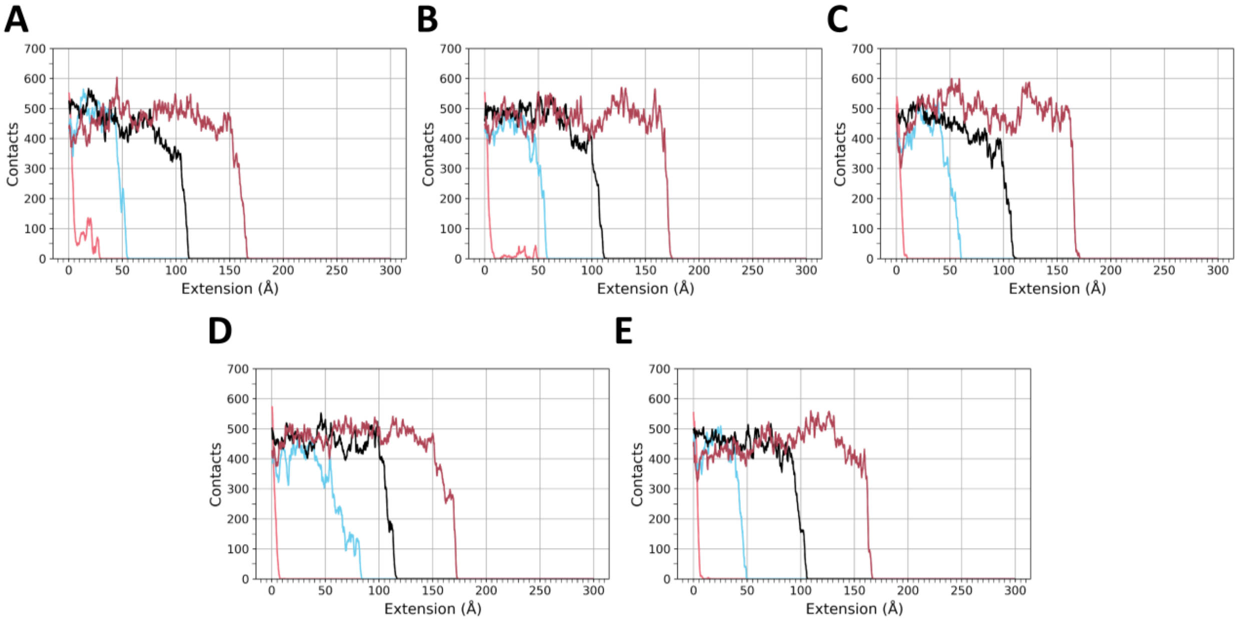

Figure S9. Total number of contacts between subunits 1 and 4 (pink), 2 and 5 (blue), 3 and 6 (black), and 4 and 7 (dark red), for the v = 5 Å/ns simulations (A) run 1, (B) run 2, (C) run 3, (D) run 4, and (E) run 5. Data are plotted as a function of extension for ease of comparison to the force vs extension curves. The contacts data are a 200 ps running average. Data are for the simulations with the staple.

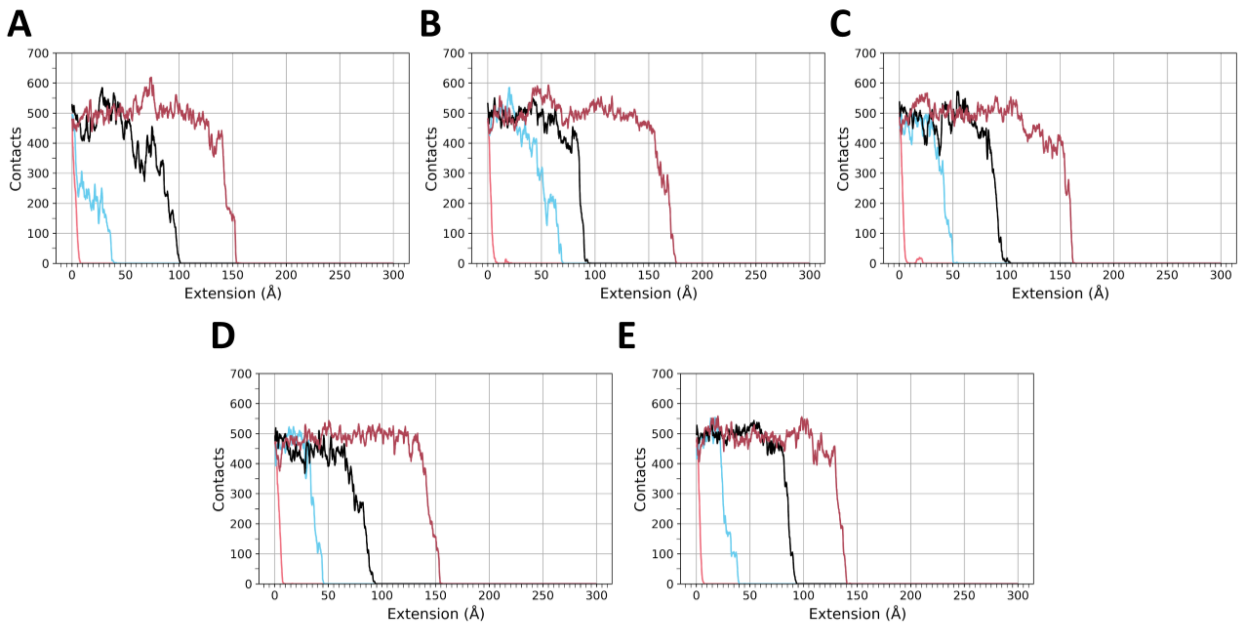

Figure S10. Total number of contacts between subunits 1 and 4 (pink), 2 and 5 (blue), 3 and 6 (black), and 4 and 7 (dark red), for the v = 5 Å/ns simulations (A) run 1, (B) run 2, (C) run 3, (D) run 4, and (E) run 5. Data are plotted as a function of extension for ease of comparison to the force vs extension curves. The contacts data are a 200 ps running average. Data are for the simulations without the staple.

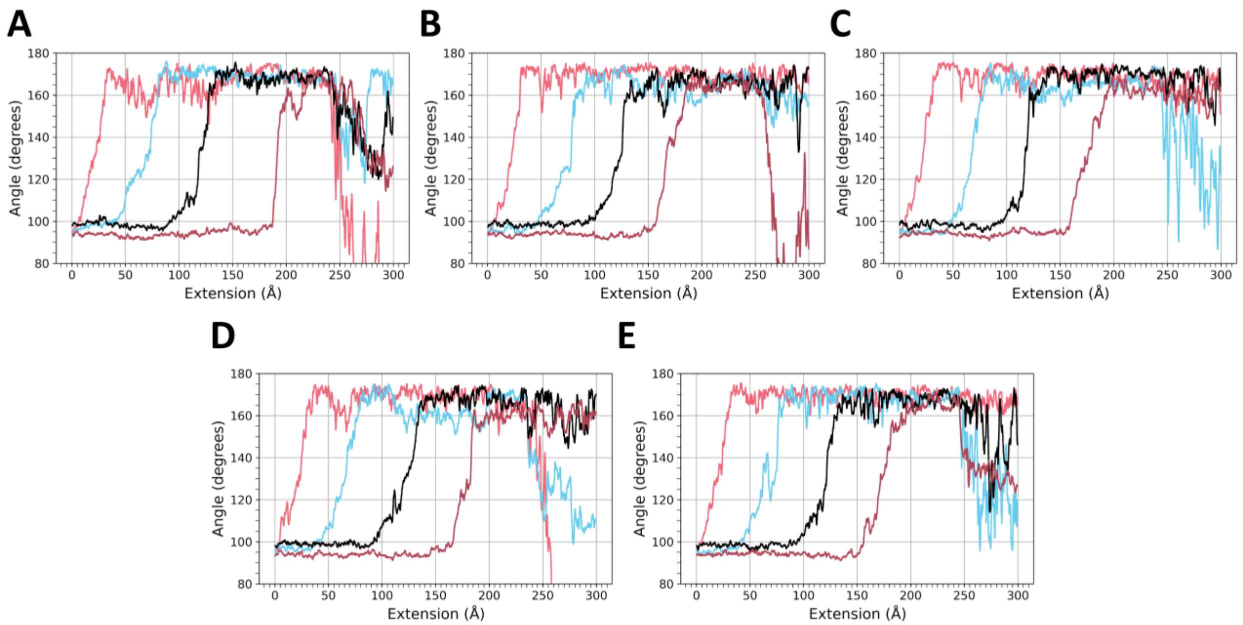

Figure S11. Angle between residues Gly7, Val18, and Cys22 (location of residues can be viewed in the main manuscript as Figure 3E, inset) for the v = 1 Å/ns simulations. As in the main manuscript Figure 3, the four curves correspond to the alignment of subunit 1 (pink curve), subunit 2 (blue curve), subunit 3 (black curve) and subunit 4 (dark red curve) with the filament axis. When the angle is 90 degrees, the subunit is perpendicular to the filament axis and when the angle is 180 degrees, the subunit is aligned with the filament axis. The angle data are a 1 ns running average. The panels correspond to (A) run 1, (B) run 2, (C) run 3, (D) run 4, and (E) run 5. Data are for the simulations with the staple.

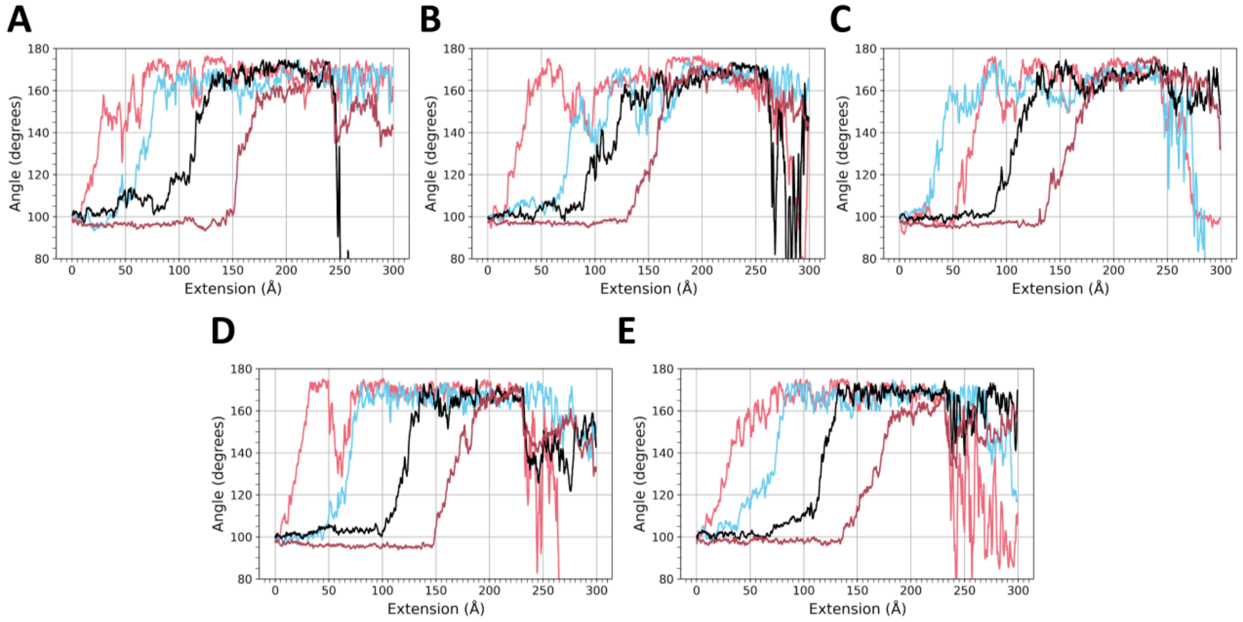

Figure S12. Angle between residues Gly7, Val18, and Cys22 (location of residues can be viewed in the main manuscript in the inset image of Figure 3E) for the v = 1 Å/ns simulations. As in the main manuscript Figure 3, the four curves correspond to the alignment of subunit 1 (pink curve), subunit 2 (blue curve), subunit 3 (black curve) and subunit 4 (dark red curve) with the filament axis. When the angle is 90 degrees, the subunit is perpendicular to the filament axis and when the angle is 180 degrees, the subunit is aligned with the filament axis. The angle data are a 1 ns running average. The panels correspond to (A) run 1, (B) run 2, (C) run 3, (D) run 4, and (E) run 5. Data are for the simulations without the staple.

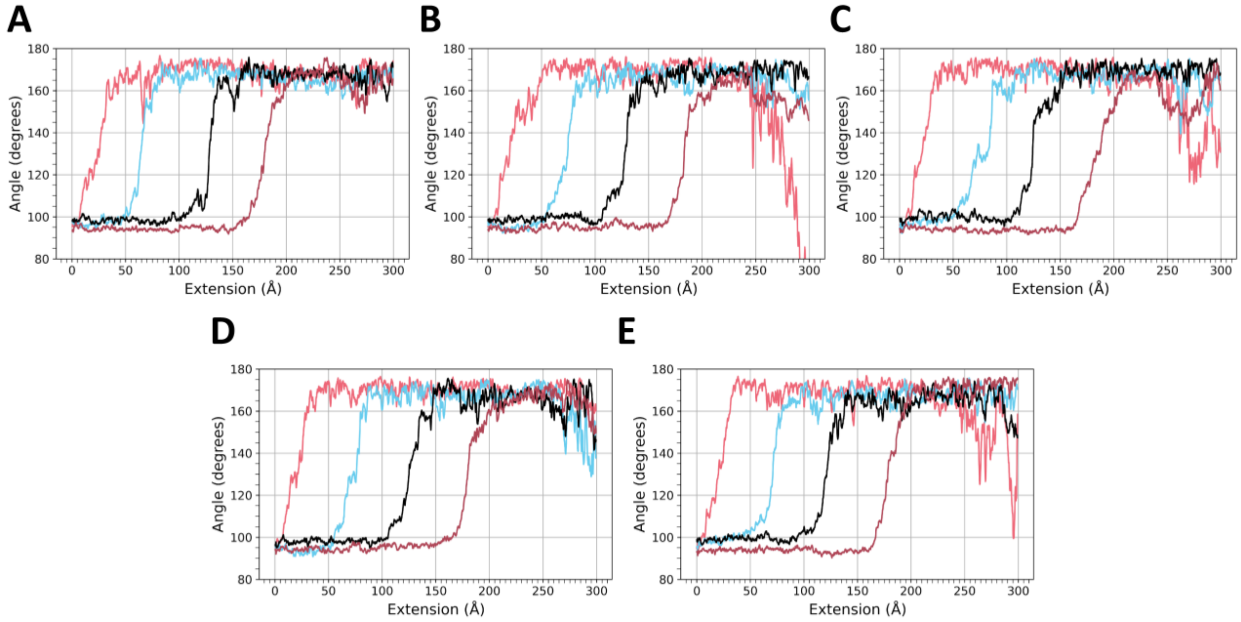

Figure S13. Angle between residues Gly7, Val18, and Cys22 (location of residues can be viewed in the main manuscript in the inset image of Figure 3E) for the v = 5 Å/ns simulations. As in the main manuscript Figure 3, the four curves correspond to the alignment of subunit 1 (pink curve), subunit 2 (blue curve), subunit 3 (black curve) and subunit 4 (dark red curve) with the filament axis. When the angle is 90 degrees, the subunit is perpendicular to the filament axis and when the angle is 180 degrees, the subunit is aligned with the filament axis. The angle data are a 200 ps running average. The panels correspond to (A) run 1, (B) run 2, (C) run 3, (D) run 4, and (E) run 5. Data are for the simulations with the staple.

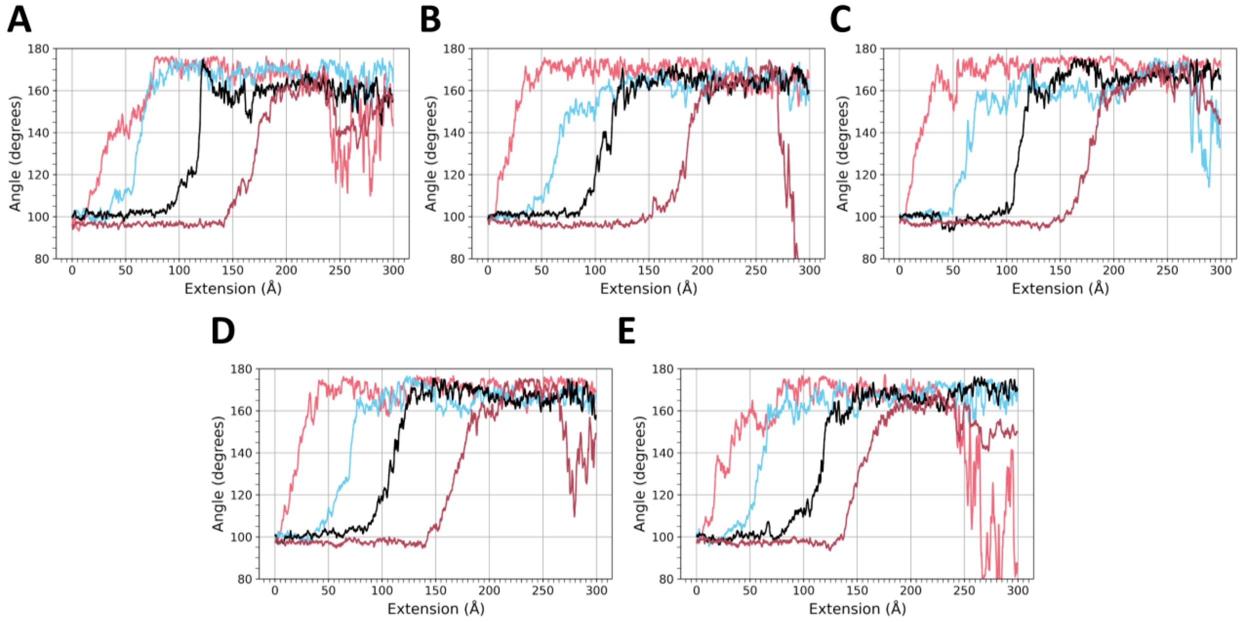

Figure S14. Angle between residues Gly7, Val18, and Cys22 (location of residues can be viewed in the main manuscript in the inset image of Figure 3 in panel E) for the v = 5 Å/ns simulations. As in the main manuscript Figure 3, the four curves correspond to the alignment of subunit 1 (pink curve), subunit 2 (blue curve), subunit 3 (black curve) and subunit 4 (dark red curve) with the filament axis. When the angle is 90 degrees, the subunit is perpendicular to the filament axis and when the angle is 180 degrees, the subunit is aligned with the filament axis. The angle data are a 200 ps running average. The panels correspond to (A) run 1, (B) run 2, (C) run 3, (D) run 4, and (E) run 5. Data are for the simulations without the staple.

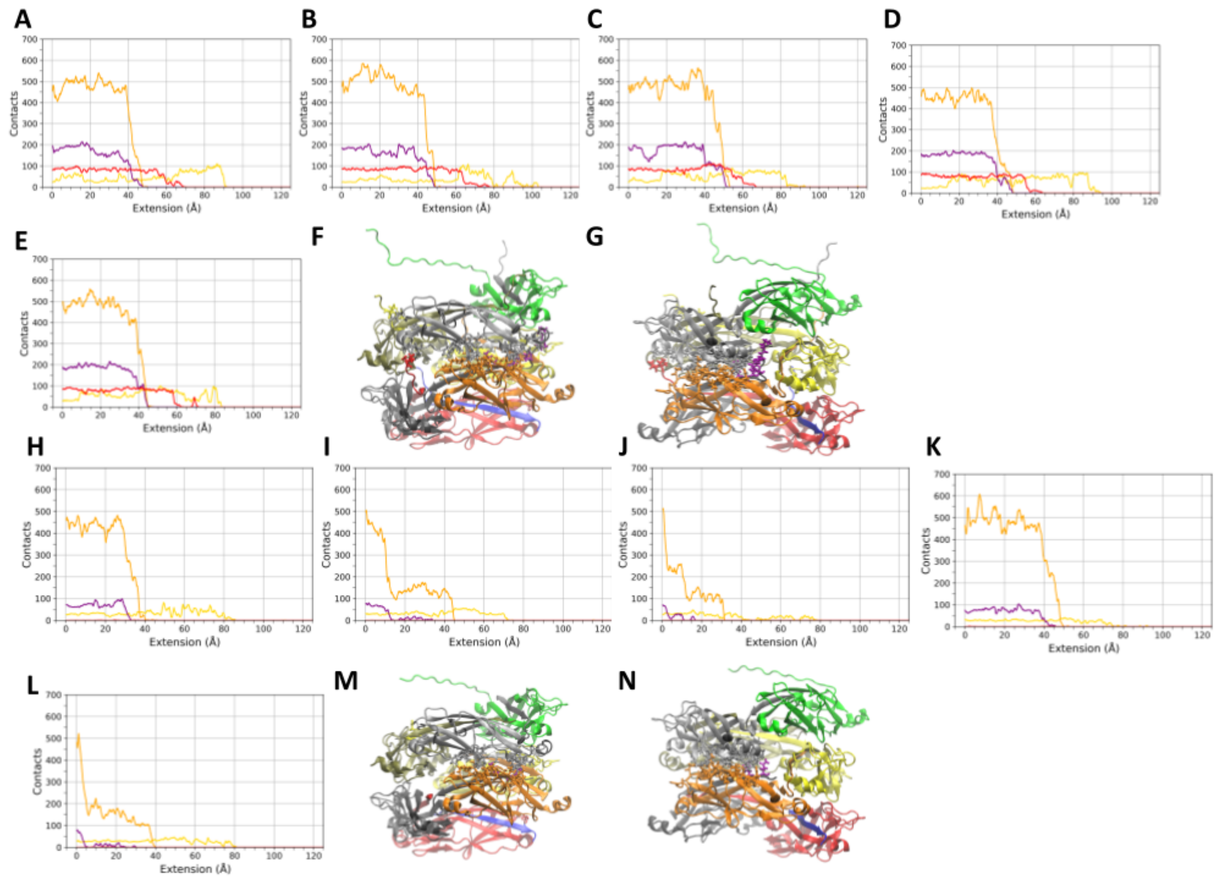

Figure S15. Total number of contacts between subunits 2 and 4 (yellow), 2 and 5 (orange), 2 and 6 (purple), and 2 and 7 (red), for the v = 1 Å/ns simulations (A) run 1, (B) run 2, (C) run 3, (D) run 4, and (E) run 5. Data are plotted as a function of extension for ease of comparison to the force vs extension curves. The plot is cut off at 125 Å since contacts between the depicted regions have all dropped to zero at that point. The contacts data are a 1 ns running average. Data in (A) - (E) are for the simulations with the staple. (F) and (G) show ribbon diagram images of the 7mer with groups of atoms that are initially within 4 Å of the light grey subunit and the yellow, orange, dark grey, and red subunits depicted in stick view. Colors of the lines in panels A through E are the same as the subunit coloring using the same color scheme as in panels F and G, except the contacts between the light grey subunit and the dark grey subunit are shown in purple for clarity. For the simulations without the staple, the total number of contacts between subunits 2 and 4 (yellow), 2 and 5 (orange), 2 and 6 (grey), and 2 and 7 (red), are shown for the v = 1 Å/ns simulations (H) run 1, (I) run 2, (J) run 3, (K) run 4, and (L) run 5. (M) and (N) show images of the 7mer without the staple with groups of atoms initially within 4 Å of the light grey subunit and the yellow, orange, dark grey, and red subunits depicted. Colors of the lines in panels (H) - (L) are the same as the subunit coloring in panels M and N, except the contacts between the light grey subunit and the dark grey subunit are shown in purple for clarity.

Contact analysis in the 100 ns equilibrium simulations

For the purposes of the data in the tables S1-S4, the presence of a contact between two residues in a frame of a trajectory is defined to be any two non-hydrogen atoms of the two residues coming within a 4 Å cutoff distance of one another. The presence of contacts was calculated at 500 ps intervals throughout the 100 ns trajectories. The time fraction is the ratio of the number of analyzed trajectory frames in which contacts are present divided by the total number of analyzed trajectory frames. Subunit-subunit contacts that occur for at least ⅓ of the equilibrium simulation time are reported. An asterisk symbol next to an "interacting residue" indicates that this interaction was also described by Hospenthal *et al.* in Figure S3 of [14].

Table S1. Contact analysis from equilibrium simulations - subunits 2 and 7
Steered molecular dynamics showing subunit-subunit contacts that occur at least ⅓ of the equilibrium simulation time between subunit 2 and subunit 7 of the 7mer filament.

Residue (Subunit 2)	Interacting residue	Interacting Subunit	Time fraction bond is present (with staple)	Time fraction bond is present (no staple)
Phe42	Pro2 *	7	0.85	Not present
Ala45	Ala1 *	7	0.71	Not present
	Pro2	7	0.93	Not present

Table S2. Contact analysis from equilibrium simulations - subunits 2 and 5
Steered molecular dynamics showing subunit-subunit contacts that occur at least ⅓ of the equilibrium simulation time between subunit 2 and subunit 5 of the 7mer filament.

Residue (Subunit 2)	Interacting residue	Interacting Subunit	Time fraction bond is present (with staple)	Time fraction bond is present (no staple)
Gln106	Asn96 *	5	1.00	0.95
	Gly97	5	0.86	0.98
	Gly98 *	5	0.62	0.86
Ala108	Pro84	5	0.62	0.69
	Asp94 *	5	0.98	0.69
	Thr95	5	1.00	0.10
	Asn96	5	0.74	0.96
Gly109	Asp94 *	5	0.74	0.57
Asn122	Gly83	5	0.58	0.29
	Pro84 *	5	0.73	0.46
Thr123	Thr82 *	5	0.88	0.9
	Asp115	5	0.61	0.62
Lys125	Thr82	5	0.82	0.69
	Phe158	5	0.98	0.94
	Asn159 *	5	1.00	0.82
Asp126	Asn159	5	1.00	0.53
Asn129	Asn157	5	0.34	0.50
Val130	Val155 *	5	0.37	0.39
	Asn157 *	5	0.97	0.99
His132	Asn96 *	5	0.81	0.96
	Ser153 *	5	0.96	0.99
	Ala154	5	0.55	0.61
	Val155 *	5	0.78	0.90
Tyr133	Asn96	5	0.97	0.75
Thr134	Asn96 *	5	1.00	0.91
	Ser153	5	0.42	0.60

Table S3. Contact analysis from equilibrium simulations - subunits 2 and 6
Steered molecular dynamics showing subunit-subunit contacts that occur at least ⅓ of the equilibrium simulation time between subunit 2 and subunit 6 of the 7mer filament.

Residue (Subunit 2)	Interacting residue	Interacting Subunit	Time fraction bond is present (with staple)	Time fraction bond is present (no staple)
Asp62	Pro2	6	0.93	Not present
	Thr3 *	6	0.98	Not present
Thr64	Thr3	6	0.59	Not present
Lys125	Gln8 *	6	1.00	0.99
Asp126	Thr3	6	0.52	Not present
	Pro5 *	6	0.68	Not present
	Gln8	6	0.42	0.41
Gly127	Pro5	6	0.59	Not present
	Gln8	6	0.96	0.66
Glu128	Gln8 *	6	1.00	0.99
Val130	Lys10	6	0.35	0.55

Table S4. Contact analysis from equilibrium simulations only found in no staple system - subunits 2 and 5
Steered molecular dynamics showing new subunit-subunit contacts that occur at least ⅓ of the equilibrium simulation time between subunit 2 and subunit 5 of the 7mer filament only in the system with no staple.

Residue (Subunit 2)	Interacting residue	Interacting Subunit	Time fraction bond is present (with staple)	Time fraction bond is present (no staple)
Asp53	Ser153	5	Not present	0.34
Lys67	Asp115	5	Not present	0.39
	Gly116	5	Not present	0.33
Gly109	Asn96	5	Not present	0.91

S3 Detailed molecular dynamics simulation methods

Preparation for steered molecular dynamics (sMD) simulations

All simulated systems were prepared using the program tLeap which is included with Amber20/AmberTools21 [5]. In the initial structure file, the 7mer pilus filament was aligned so that the helical axis was along the z-direction. This orientation was used for all simulations. Note that for the subunit at the “base” of the filament, the inserted β strand from the prior subunit is also included in the simulated structure (using the first 20 amino acids of the N-terminal extension (NTE)). Protein parameters were described by the FF14SB force field [6] and the parameters for water molecules were described by the TIP3P force field [7]. Monovalent counterions were described using the parameters of Joung and Cheatham [8].

The 7mer systems were solvated in a rectangular water box. In the x and y directions (perpendicular to the filament axis) a 12 Å buffer to the periodic cell edge was used, and in the z-direction (parallel to the filament axis) a 160 Å buffer was implemented. This allowed for the filament to be extended along the filament axis during sMD simulations while maintaining its solvation. Overall charge neutralization was achieved by adding 35 Na^+ counterions to the system.

Preparation of the systems for sMD simulations used an approach very similar to that in simulations of the 3mer system [9], but modified accordingly to simulate the larger 7mer system for the current study. Differences in protocol between simulations with and without the staple are pointed out specifically in the text below. Note that for simulations without the staple, the first five amino acids were removed from the N-terminal end of each subunit in the 7mer system, so that the first amino acid in each subunit becomes Gln6. Energy minimization was accomplished using 3000 steps of steepest descent and 2000 steps of conjugate gradient while applying a restraint force constant of 10.0 kcal mol^{-1} $Å^{-2}$ to the alpha carbons. Subsequently, system heating was carried out using two separate stages. First, the system temperature was increased from 0 K to 100 K in the NVT ensemble over 20 ps, and then the temperature was held at 100 K for 30 ps. Second, heating using the NPT ensemble was performed and the system temperature was increased from 100 K to 300 K (20 ps duration) and then held at a temperature of 300 K (80 ps duration). During the heating stages, a restraint force constant of 10.0 kcal mol^{-1} $Å^{-2}$ was applied to the alpha carbons. After heating, equilibration of the systems was carried out over seven stages while maintaining a temperature of 300 K in the NPT ensemble, and using a

changing set of atom restraints depending on the equilibration stage. Table S5 below describes how the restraints were applied during the equilibration stages of simulation. After the equilibration was completed, the last frame was used as the initial coordinates for the sMD simulations.

Table S5. Restraints during equilibration stages to prepare for sMD simulations

Stage	Duration (ps)	Restrained selection	Restraint strength (kcal mol^{-1} $Å^{-2}$)
1	200	All alpha carbons	10.0
2	200	All alpha carbons	5.0
3	200	All alpha carbons	2.5
4	200	All alpha carbons	0.5
5	200	All alpha carbons	0.1
6	1000	Alpha carbons of Subunit 7 (and its inserted NTE β-strand from Subunit 8), Subunit 6, Subunit 5 (except for its NTE β-strand)	0.1
7	4000	Alpha carbons of Subunit 7 (and its inserted NTE β-strand from Subunit 8), Subunit 6, Subunit 5 (except for its NTE β-strand), Subunit 1	0.1

sMD simulations

To implement the sMD protocol, the jar = 1 [10] flag in Amber20 was set and all of the sMD simulations were carried out using the NPT ensemble with a temperature of 300 K. The sMD simulations require the definition of a collective variable (CV) used to describe the extension of the system. We used the z-distance between the center of mass of the alpha carbons of subunits 7 (and its inserted NTE β-strand from Subunit 8), subunit 6, and subunit 5 (except for its NTE β-strand), and the alpha carbons of the terminal (1st) subunit in the 7mer (except for the alpha carbons in its NTE β-strand to allow that region to remain freely mobile during 7mer extension). We refer to those selections as the “fixed” and “pulled” selections, respectively. In order to apply the sMD force along the z-direction, the AMBER20 fxyz option was used. The fixed selection was restrained by applying a 0.5 kcal mol^{-1} $Å^{-2}$ restraint to the alpha carbons in that atom group. This allowed us to have the bottom three subunits mimic a segment of the filament base being adhered to a surface as the top subunit is pulled, allowing four out of the seven subunits to extend away from the base in the sMD simulations. The restraints applied to the three subunits at the base also eliminated the possibility for overall rotations and translations of the system during the sMD simulations. Systems were extended by approximately 300 Å along the z-direction, and this led to overall elongation of the 7mer system, and in some cases eventually led to breakage of the system between subunits (.e.g, separation by the extraction of an NTE β-strand). The spring constant parameter for the pulling spring in the AMBER input files was set to a value of 5.0, which results in a pulling spring stiffness of 10.0 kcal mol^{-1} $Å^{-2}$. The force and amount of extension were saved every 2 ps. During the heating, equilibration, and sMD simulations the integration timestep was set to 2 fs, and the SHAKE algorithm was applied

to hydrogen bonds [11]. The Langevin thermostat was used for temperature control, and we implemented a collision frequency of 1 ps^{-1}. During stages of the simulation that were carried out in the NPT ensemble, the Monte Carlo barostat [12] was used to maintain a pressure of 1 atm. All stages of simulation employed a real space interaction cutoff of 8 Å, and long range electrostatics were handled using the particle mesh Ewald method [13].

Extended equilibrium simulations

We also carried out two extended equilibrium simulations of the 7mer filaments, one for the system with the staple residues, and one for the simulation of the system without the staple residues. The equilibrium simulations lasted for 100 ns. They were carried out in the NPT ensemble, again using the Langevin thermostat and the Monte Carlo barostat as described above to maintain a temperature of 300 K and a pressure of 1 atm. During these simulations, restraints were only applied to the alpha carbons of the bottom three subunits of each system as described in Table S5 above, with the following modification: the first 8 amino acids of the subunit 8 NTE β-strand, as well as the first 8 amino acids of subunits 7 and 6, were allowed to remain unrestrained (without the staple this was residues 6-8, as 1-5 were deleted). Neither long equilibrium simulation had restraints applied to the terminal (1st) subunit in the 7mer filament. The restraints applied to the alpha carbon atoms of the bottom three subunits prevented overall translation and rotation of the 7mer during the extended equilibrium simulations.

Steered Molecular Dynamics Movie Captions

Movie S1. Movie generated in VMD of the v = 1 Å/ns steered molecular dynamics simulations of the P pilus system with the staple for runs 1 through run 5.

Movie S2. Movie generated in VMD of the v = 1 Å/ns steered molecular dynamics simulations of the P pilus system without the staple for runs 1 through run 5.

Movie S3. Movie generated in VMD of the v = 5 Å/ns steered molecular dynamics simulations of the P pilus system with the staple for runs 1 through run 5.

Movie S4. Movie generated in VMD of the v = 5 Å/ns steered molecular dynamics simulations of the P pilus system without the staple for runs 1 through run 5.

References

1. Capitanio, Marco, and Francesco S Pavone, Interrogating biology with force: single molecule high-resolution measurements with optical tweezers, Biophysical journal vol. 105,6 1293-303 (2013)

2. Odijk, T. . Stiff chains and filaments under tension. Macromolecules, 28(20), 7016-7018 (1995)

3. Flory, P. J., & Volkenstein, M. Statistical mechanics of chain molecules (1969)

4. O. Björnham, O. Axner, M. Andersson, Modeling of the elongation and retraction of Escherichia coli P pili under strain by Monte Carlo simulations, European Biophysics Journal 37, 381–391 (2008)

5. D. A. Case, et al., AMBER 2021. Univ. California, San Fr. (2021).

6. J. A. Maier, et al., ff14SB: Improving the Accuracy of Protein Side Chain and Backbone Parameters from ff99SB. J. Chem. Theory Comput. 11, 3696–3713 (2015).

7. W. L. Jorgensen, J. Chandrasekhar, J. D. Madura, R. W. Impey, M. L. Klein, Comparison of simple potential functions for simulating liquid water. J. Chem. Phys. 79, 926–935 (1983).

8. I. S. Joung, T. E. Cheatham, Determination of alkali and halide monovalent ion parameters for use in explicitly solvated biomolecular simulations. J. Phys. Chem. B 112, 9020–9041 (2008).

9. Baker, J. L., Dahlberg, T., Bullitt, E., & Andersson, M. Impact of an alpha helix and a cysteine–cysteine disulfide bond on the resistance of bacterial adhesion pili to stress. Proceedings of the National Academy of Sciences, 118(21), e2023595118. (2021)

10. A. Crespo, M. A. Martí, D. A. Estrin, A. E. Roitberg, Multiple-steering QM− MM calculation of the free energy profile in chorismate mutase. J. Am. Chem. Soc. 127, 6940–6941 (2005).

11. J.-P. Ryckaert, G. Ciccotti, H. J. C. Berendsen, Numerical integration of the cartesian equations of motion of a system with constraints: molecular dynamics of n-alkanes. J. Comput. Phys. 23, 327–341 (1977).

12. Allen, M. P., & Tildesley, D. J. Computer simulation of liquids. Oxford university press (2017)

13. Salomon-Ferrer, R., Götz, A. W., Poole, D., Le Grand, S., & Walker, R. C. . Routine microsecond molecular dynamics simulations with AMBER on GPUs. 2. Explicit solvent particle mesh Ewald. Journal of Chemical Theory and Computation, *9*(9), 3878–3888 (2013)

14. Hospenthal, M. K., Redzej, A., Dodson, K., Ukleja, M., Frenz, B., Rodrigues, C., Hultgren, S. J., DiMaio, F., Egelman, E. H., & Waksman, G. (2016). Structure of a Chaperone-Usher Pilus Reveals the Molecular Basis of Rod Uncoiling. Cell, 164(1–2), 269–278.
